# Class II contact‐dependent growth inhibition (CDI) systems allow for broad‐range cross‐species toxin delivery within the *Enterobacteriaceae* family

**DOI:** 10.1111/mmi.14214

**Published:** 2019-03-18

**Authors:** Petra Virtanen, Marcus Wäneskog, Sanna Koskiniemi

**Affiliations:** ^1^ Department of Cell and Molecular Biology Uppsala University Uppsala 75124 Sweden

## Abstract

Contact‐dependent growth inhibition (CDI) allows bacteria to recognize kin cells in mixed bacterial populations. In *Escherichia coli*, CDI mediated effector delivery has been shown to be species‐specific, with a preference for the own strain over others. This specificity is achieved through an interaction between a receptor‐binding domain in the CdiA protein and its cognate receptor protein on the target cell. But how conserved this specificity is has not previously been investigated in detail. Here, we show that class II CdiA receptor‐binding domains and their *Enterobacter cloacae *analog are highly promiscuous, and can allow for efficient effector delivery into several different *Enterobacteriaceae *species, including *Escherichia,* *Enterobacter,* *Klebsiella and Salmonella* spp. In addition, although we observe a preference for the own receptors over others for two of the receptor‐binding domains, this did not limit cross‐species effector delivery in all experimental conditions. These results suggest that class II CdiA proteins could allow for broad‐range and cross‐species growth inhibition in mixed bacterial populations.

## Graphical abstract

Contact‐dependent growth inhibition (CDI) has previously been thought to be species‐specific. Here we correct that misconception and show that CDI can be highly promiscuous and allow for cross‐species toxin delivery to a wide range of *Enterobacteriaceae*. Furthermore, we show that this promiscuous CDI activity is dependent on the environmental context, as we observe a preference for intra‐species CDI interactions in liquid media but not on solid media.

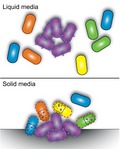

## Introduction

Bacteria live in complex microbial communities where they interact and compete with other microbes for nutrients. In order to take up certain nutrients, e.g. iron, bacteria must first secrete proteins that sequester iron, i.e. siderophores to the extra‐cellular milieu. These siderophores are considered shared goods, and can be taken up by any bacteria with the correct receptor, i.e. most *Enterobacterieaceae*. To benefit from the use of shared goods, it is important to limit the use of these goods to one’s own kin. To that end, bacteria use a number of different strategies, including toxin delivery systems for non‐self‐exclusion to ensure that the cells closest to the producer are of the own kin (Wall, 2016). Delivery of antibacterial toxins to neighboring cells can occur through different mechanisms, including cell–cell contact mediated effector delivery by type IV (Souza *et al*., 2015), V (Aoki *et al*., 2005), VI (Hood *et al*., 2010) and VII (Cao *et al*., 2016) secretion systems. Common for all these toxin delivery systems is that kin cells express an immunity or antitoxin protein that protects against the toxic activity of its cognate effector, allowing kin cells to survive effector delivery when non‐kin cells are killed or growth inhibited (Aoki *et al*., 2005; 2010; Hood *et al*., 2010; Russell *et al*., 2014; Cao *et al*., 2016).

Contact‐dependent growth inhibition (CDI) systems belong to the type 5 secretion system (T5SS) subclass B, also known as two‐partner secretion (TPS) systems (Hayes *et al*., 2010). During CDI, the β‐barrel CdiB protein exports and presents the ‘stick‐like’ CdiA protein on the cell‐surface (Leo *et al*., 2012; Ruhe *et al*., 2013a; 2018). The delivered toxic effector is encoded in the C‐terminal domain of the CdiA protein (CdiA‐CT). Upon interaction with a receptor on the cell surface of a neighboring cell, the C‐terminal domain is cleaved off and delivered into the recipient cell (Aoki *et al*., 2010; Webb *et al*., 2013; Ruhe *et al*., 2018). The toxic activities of the C‐terminal encoded toxic effectors range from nucleases that degrade tRNA, rRNA and DNA to ionophore toxins that dissipate the proton motive force of the bacterial cell (Aoki *et al*., 2009; 2010; Morse *et al*., 2012). To protect themselves from auto‐inhibition bacteria with CdiA toxins express a small CdiI immunity protein, which binds and forms a tight complex with its cognate toxin, neutralizing its toxic activity (Aoki *et al*., 2005; Morse *et al*., 2012).

*Escherichia coli* CdiA proteins can be divided into different classes (Class I–V) based on sequence homology. The sequence variation between Class I, II and III CdiA proteins is mainly found in the receptor‐binding domain (RBD), with the exception of the toxic C‐terminal domains (which are highly variable as they encode for toxins with diverse toxic activities). In the first identified Class I CdiA protein of *E. coli* 93 the RBD is found in the middle of the CdiA protein (residues ~1300–1600aa and ~1900–2300aa) (Ruhe *et al*., 2017). These CdiA RBD interact with different outer‐membrane proteins on the surface of targeted cells; BamA for Class I (Aoki *et al*., 2008; Ruhe *et al*., 2013b), OmpC‐OmpF for Class II (Beck *et al*., 2016) and Stx for Class III (Ruhe *et al*., 2017), enabling delivery of effectors to the recipient cell. Previous studies suggest that effector delivery by class I CdiA proteins is strictly species‐specific and limited to *E. coli* (Ruhe *et al*., 2013b) and that the class II CdiA proteins are strain‐specific and able to discriminate between different strains of *E. coli,* with a preference for the ‘own’ strain over others (Beck *et al*., 2016). This specificity is achieved by differences in the protein sequences of the extracellular loops of the receptors, which presumably affects the binding affinity between the receptor and the RBD of CdiA. After being delivered through the outer membrane of a target cell, the C‐terminal toxin domains of many CdiA toxins associate with different inner‐membrane proteins for translocation into or through the inner‐membrane (depending on the activity of the toxin). The latter interaction does not seem species‐specific as toxins from many different species can be attached to the CdiA‐stick and be efficiently delivered into *E. coli* cells (Willett *et al*., 2015).

We were interested in finding out more about the specificity of binding between class II CdiA RBD and their cognate receptors, hoping to learn more about the role that CDI systems play in kin‐recognition. Previous studies on kin recognition proteins like TraA in *Myxococus xanthus,* show that single amino acid changes are sufficient for differential binding between proteins and their cognate receptors (Cao and Wall, 2017) and we wanted to investigate if this is also the case for the interaction between CdiA and the OmpC component of the receptor, whose extracellular loops have previously been shown to drive specificity (Beck *et al*., 2016). We used bioinformatics to find similar but not identical CdiA RBD to investigate if small changes in the RBD changed the receptor recognition and thus the species‐specificity of CdiA toxin delivery. Somewhat surprisingly, we find that a *E. coli* class II CdiA RBD allow for delivery of toxic effectors into many different *Enterobacteriaceae* spp., including* Enterobacter* *cloacae *and *aerogenes,* *Klebsiella pneumoniae *and *Salmonella typhimurium,* suggesting that class II CDI is a broad‐range inter‐species competition system. Additionally, two class II CdiA RBD homologs and an *E. cloacae *analog, allow effector delivery to cells expressing non‐cognate OmpC receptors. Furthermore, even though our results suggest that CdiA‐receptor binding correlates well with inhibition during continuous agitation, strong receptor binding is not required for CDI on solid media. Taken together, our results suggest that class II CdiA molecules could allow for kin recognition through inhibition of other bacteria and at the same time illustrate how the versatility of CDI as a competition and kin recognition system changes, depending on the environmental context.

## Results

### Identification of CdiA proteins in Enterobacteriaceae spp.

To increase our understanding of the interaction between the class II CdiA RBD and its cognate OmpC receptor, we set out to bioinformatically identify similar but not identical CdiA receptor‐binding domains with the intention of studying if small changes in the receptor‐binding domain affects receptor‐specificity. Our bioinformatic analyses identified identical and closely homologous class II CdiA receptor‐binding domains in *E. coli* strains with quite different OmpC protein sequences. For example, *E. coli *UPEC F11 has an identical CdiA binding domain to that found in the previously studied UPEC 536 (Fig. S1) but has very different extracellular loops of OmpC (Fig. S2). In addition, closely homologous binding domains (few aa differences) to the CdiA protein of *E. coli* UPEC F11 (CdiA^F11^) were identified in *Salmonella* *typhi,* *E. coli* CFT073/*E. coli* Nissle 1917 (Fig. S1), which also have significantly different OmpC extracellular loops from UPEC 536, UPEC F11 and each other (with the exception of *E. coli* CFT073 and Nissle 1917 where both binding domains and OmpC sequences were identical) (Fig. S2)*.* Thus, these findings suggest that species‐specificity could be achieved by very small amino acid differences in the receptor and/or receptor‐binding domain.

### Class II CdiA‐OmpC dependent effector delivery is promiscuous

**Figure 1:**
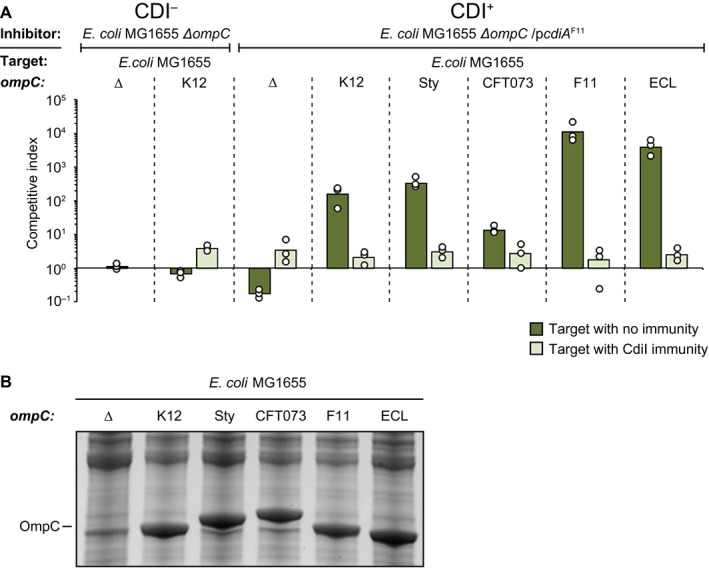
Class II CdiA proteins are able to deliver effectors to cells expressing the OmpC receptor from other strains and species. A. Average competitive index of cells expressing CdiA^F11^ from a ColE1 plasmid after co‐culturing with MG1655 cells expressing different OmpC’s from the native MG1655 *ompC *locus with (light green bars) or without (dark green bars) CdiI expressed from a CloDF plasmid (*n* = 3 biological replicates). Cells were co‐cultured for 5 h in liquid LB media. Individual data points of the biological replicates are shown as black and white circles. B. MG1655 cells expressing different OmpC’s from the native MG1655 *ompC *locus from Fig. 1A were grown in LB and outer‐membrane fractions were enriched and separated by SDS‐PAGE. [Colour figure can be viewed at https://www.wileyonlinelibrary.com]

To test how the differences between OmpC proteins affected class II mediated toxin delivery, we replaced the chromosomal *ompC *ORF of *E. coli* MG1655 with the *ompC* from *E. coli* strains UPEC F11 or Nissle 1917/CFT073, as well as the *ompC* from *E. cloacae* and *S. typhimurium/typhi *(OmpC’s from *S. typhimurium* and *typhi* are identical)*. *Next, we competed these strains with an *E. coli* MG1655 strain expressing a chimeric CdiA protein with the receptor‐binding domain from UPEC F11 (CdiA^F11^) from a medium copy (ColE1) plasmid containing the *cdiBAI *locus from EC93 under its native promoter. Surprisingly, cells expressing CdiA^F11 ^outcompeted all target strains during co‐cultivation in liquid LB media (Fig. 1A, dark green bars). Inhibition varied between 4‐logs for the *E. coli* UPEC F11 and *E. cloacae *OmpC’s (OmpC^F11 ^and OmpC^ECL^ respectively) to 1‐log for the OmpC of CFT073 (OmpC^CFT073^) (Fig. 1A, dark green bars). Cells expressing OmpC variants from *S. typhimurium/typhi *(OmpC*^Sty^*), or the native OmpC of *E. coli* MG1655 (OmpC^K12^) were outcompeted by 2‐logs (Fig. 1A, dark green bars). Furthermore, cells expressing CdiA^F11 ^were not able to outcompete cells expressing CdiI immunity protein irrespective of their OmpC, suggesting that the observed ability to outcompete was indeed mediated by toxic effector delivery into the different strains (Fig. 1A, light green bars). To further confirm that the observed growth inhibition was due to toxin delivery, we used cells lacking the *ompC* gene (∆*ompC). *MG1655 cells lacking *ompC* were not outcompeted by cells expressing CdiA^F11 ^(Fig. 1A), further confirming that the observed inhibition was mediated by CDI and that OmpC indeed functions as a receptor for CdiA^F11^. Notably, expression of *cdiI* came with a similar fitness cost for the cells as expressing *cdiBAI*.

We did observe differences in the level of inhibition between cells expressing different OmpC variants. There are two possible explanations to this observation, (i) some OmpC’s allow more efficient toxin delivery or (ii) the target strains could express different levels of the OmpC proteins. To test the latter we used SYPRO Ruby (Thermo Scientific) protein staining to measure OmpC levels in the different strains. OmpC protein levels were very similar in the different target strains (Fig. 1B), suggesting that OmpC levels are not the reason for the difference in inhibition between these strains. Taken together, our results suggest that class II CdiA mediated effector delivery is not species‐specific, but that there could be a difference in class II CdiA receptor specificity.

### OmpC levels are important for *cdiBAI *mediated growth inhibition

**Figure 2:**
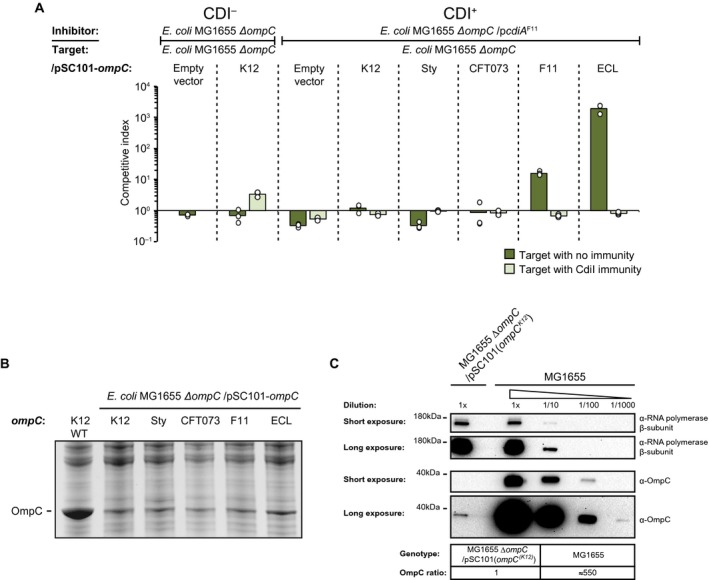
Class II CdiA toxin delivery is affected by OmpC expression levels. A. Average competitive index of cells expressing CdiA^F11^ after co‐culturing with MG1655 cells expressing different OmpC’s from a low‐copy (pSC101) plasmid with (light green bars) or without (dark green bars) CdiI expressed from plasmid (*n*=3 biological replicates). Cells were co‐cultured for 5 h in liquid LB media. Individual data points of the biological replicates are shown as black and white circles. B. MG1655 cells expressing different OmpC’s from Fig. 2A were grown in LB and outer membrane fractions were enriched and separated by SDS‐PAGE. C. Western blot of MG1655 cells from Fig. 1A and 2A expressing OmpC^K12^ either from the chromosome or from the low‐copy (pSC101) plasmid. [Colour figure can be viewed at https://www.wileyonlinelibrary.com]

Our findings of class II CdiA cross‐species growth inhibition are in contrast with previous findings where cells expressing a class II CdiA protein from UPEC 536 (CdiA^UPEC^) were not able to deliver effectors to *E. coli* cells expressing OmpC from *S. typhimurium *(Beck *et al*., 2016). The RBD of UPEC 536 and F11 are identical, but to our surprise, MG1655 strains expressing OmpC*^Sty^* were inhibited as efficiently as wild type MG1655 cells (OmpC^K12^) by inhibitor cells expressing CdiA^F11^. In the previous study, a plasmid‐based construct was used to express OmpC*^Sty^* from an uninduced, leaky pTac promoter resulting in OmpC levels that are similar to natively expressed OmpF levels (Beck *et al*., 2016), which according to our data are very low in these conditions (undetectable by western blot, see Fig. 5B). Our construct has the *ompC* ORF from *S. typhimurium/typhi *inserted on the chromosome under the native *ompC* promoter and should, under these conditions, express roughly 100,000 OmpC molecules/cell (Schuman, 2006). Thus, an obvious difference between these constructs is the expression level of OmpC. To test if OmpC expression levels are important for CdiA cross‐species effector delivery, we cloned all the tested *ompC* ORFs onto a low‐copy (pSC101) plasmid backbone, to be expressed from a synthetic, medium strong, constitutive promoter; PJ23101 (Kelly *et al*., 2009). Cells expressing CdiA^F11^ were able to outcompete MG1655 cells expressing low levels of OmpC^F11^ or OmpC^ECL^ in liquid LB media, whereas target cells expressing any of the other OmpC variants were not inhibited (Fig. 2A). This is different from the growth inhibition observed of strains expressing the chromosomally encoded OmpC’s, where all target cells, regardless of OmpC variant, were inhibited (Fig. 1A). These results are in line with the previously reported lack of inhibition of target cells expressing OmpC*^Sty^* (Beck *et al*., 2016). We confirmed that our plasmid constructs expressed lower levels of OmpC than our chromosomal constructs using SDS‐PAGE (Fig. 2B) and western blot (Fig. 2C). The chromosomal constructs expressed ~550‐fold more OmpC than the plasmid constructs during growth in liquid LB media (Fig. 2C). Thus, the level of the OmpC receptor does affect effector delivery, which could be the reason why a broad‐range cross‐species effector delivery has not previously been reported.

### Inhibition correlates with CdiA‐receptor binding

**Figure 3:**
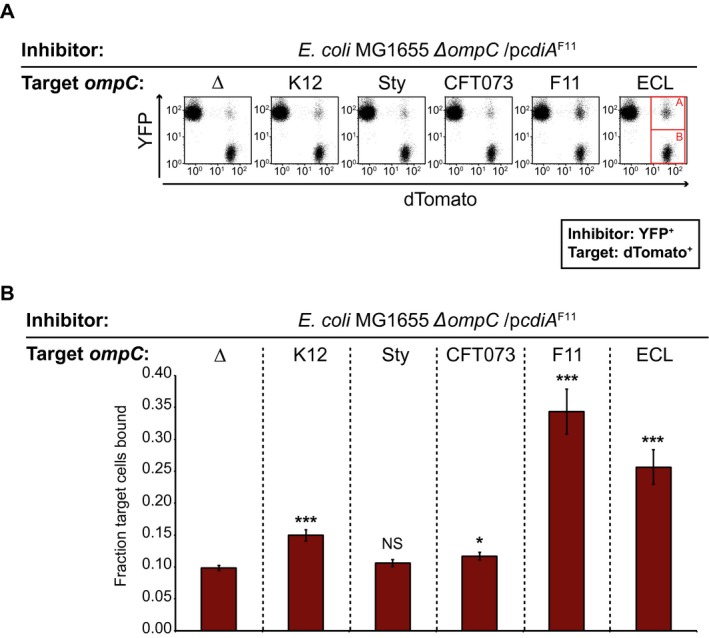
CdiA‐OmpC mediated cell‐cell binding. YFP+ MG1655 cells expressing CdiA^F11^ were mixed with dTomato+ MG1655 cells expressing none (*ΔompC*) or different OmpC’s from the native *ompC* locus on the MG1655 chromosome. Cell‐cell binding was analyzed by flow cytometry. A. Representative images of flow cytometry cell‐cell binding assay. Bound dTomato+ target cells (A, red square) were divided by total number of dTomato+ cells (A+B, red squares) to calculate fraction of bound target cells. B. Average fraction of MG1655 target cells bound to CdiA^F11^ expressing inhibitor cells (*n* = 6 biological replicates). Error‐bars are SEM. Statistical significance was determined using a two‐tailed, unpaired student’s *t*‐test where * = *P* < 0.05, ** = *P* < 0.005 and *** = *P *< 0.0005. [Colour figure can be viewed at https://www.wileyonlinelibrary.com]

Our results suggest that cells expressing CdiA^F11^ proteins are able to inhibit the growth of cells expressing OmpC from F11 and *E. cloacae* more than cells expressing other OmpC variants. This does not necessarily mean that the binding interactions between the CdiA and the different OmpC proteins vary. To test if CdiA proteins with the class II binding domain have different binding affinity for OmpC’s from different species, we used a previously described cell–cell binding assay (Aoki *et al*., 2008). In short, inhibitor cells expressing CdiA^F11 ^were modified to constitutively express one fluorophore (sYFP2) and target cells with different OmpC variants (OmpC^K12^, OmpC^Sty^, OmpC^CFT073^, OmpC^F11^ or OmpC^ECL^) expressing another fluorophore (dTomato). Inhibitor and target cells were mixed and after 40 min of co‐cultivation at a high cell‐density in liquid LB with shaking the fraction of dTomato^+^ target cells (with different OmpC variants) bound to sYFP2^+^ inhibitor cells (with CdiA^F11^) were analyzed by flow cytometry (Fig. 3A). To control for receptor independent binding target cells lacking the OmpC receptor were used. Approximately 34% of target cells expressing OmpC^F11^ were bound to cells expressing CdiA^F11^, compared to 26% of cells expressing OmpC^ECL^, 15% of cells expressing OmpC^K12^ and 12% of cells expressing OmpC^CFT073^(Fig. 3A and B). Around 10% of *ΔompC* target cells were bound to inhibitors (receptor independent cell‐cell interactions) (Fig. 3B). For target cells expressing OmpC^Sty^, binding above background levels (10%) could not be detected, even though these cells were inhibited to the same extent as cells expressing OmpC ^K12^ (Fig. 1A).

**Figure 4:**
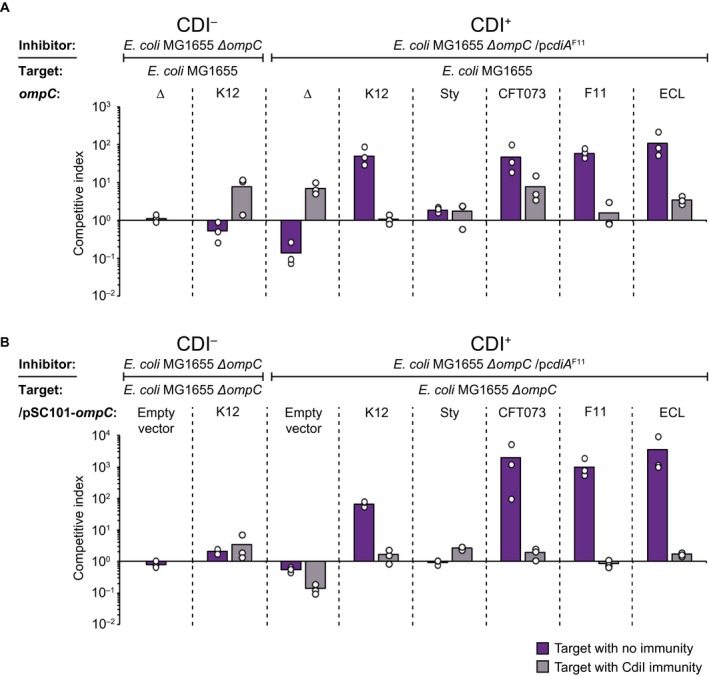
Growth on solid media overcomes weak CdiA‐OmpC binding. Average competitive index of cells expressing CdiA^F11^ from a ColE1 plasmid after co‐culturing with MG1655 cells expressing different OmpC’s from the native MG1655 *ompC *locus (A) or from the low‐copy (pSC101) plasmid (B) with (light grey bars) or without (dark purple bars) CdiI expressed from a pCloDF plasmid (*n* = 3 biological replicates). Cells were co‐cultured for 24 h on solid M9Glu media. Individual data points of the biological replicates are shown as black and white circles. [Colour figure can be viewed at https://www.wileyonlinelibrary.com]

We correlated relative cell–cell binding versus CDI mediated growth inhibition and could observe that there was an almost perfect correlation (*P* < 0.01) between cell‐cell binding and growth inhibition (Fig. S3). However, while OmpC^Sty ^did not support any significant cell–cell binding we could still observe a strong growth inhibition (Fig. 1A). Thus, we concluded that strong cell–cell binding is not always beneficial for CDI mediated growth inhibition. As our binding assay measures steady‐state receptor binding both CdiA‐OmpC association and dissociation are measured in our assay (Fig. 3A and B). Thus, our results could be explained by CdiA^F11^‐OmpC^Sty^ receptor binding not following the same trend with regards to receptor association and/or receptor disassociation as the other OmpC variants investigated in this study. Nevertheless, this relationship between cell–cell binding and growth inhibition is well described by a polynomial distribution and thus we concluded that the measured CdiA‐OmpC interaction does correlate with observed growth inhibition in this context.

### Growth on solid media overcomes weak CdiA‐OmpC binding

In their natural environment, most bacteria grow on solid surfaces or within biofilms. In such conditions, the binding affinity between CdiA and its cognate receptor might not play a significant role, as binding is not required to keep proximity to the neighboring cell. To test this, we competed MG1655 cells expressing CdiA^F11^ against MG1655 cells with low (plasmid) or high (chromosomal) expression of the different OmpC variants on solid rich defined M9 media supplemented with glucose and casamino acids (M9Glu) (for details regarding the media see the materials section). MG1655 cells expressing high levels of the different OmpC variants were inhibited similarly; around 2‐log for all OmpC variants except OmpC*^Sty ^*where no inhibition could be seen (Fig. 4A). Similarly, MG1655 cells expressing low levels of any of the OmpC variants except OmpC*^Sty^, *were also outcompeted with 2‐ to 3‐logs on solid media (Fig. 4B). Notably the fitness cost of carrying the CDI system (as observed by the CdiA^F11^ vs. Δ*ompC* competitions) was almost abolished by the presence of the pSC101 OmpC expression plasmid in the target strains (Fig. 4B), whereas the same cost added more than 1‐log of difference to the strains lacking the pSC101 OmpC expression plasmid (∆*ompC*, Fig. 4A). Taken this into account, there was not much difference in the level of inhibition between strains expressing high or low levels of the receptor when competed on solid media (Fig. 4A and B), suggesting that neither low receptor abundance nor weak CdiA‐OmpC binding affinity prevent or limit growth inhibition on solid media.

### OmpF is beneficial but not essential for CdiA^F11^ mediated growth inhibition

**Figure 5:**
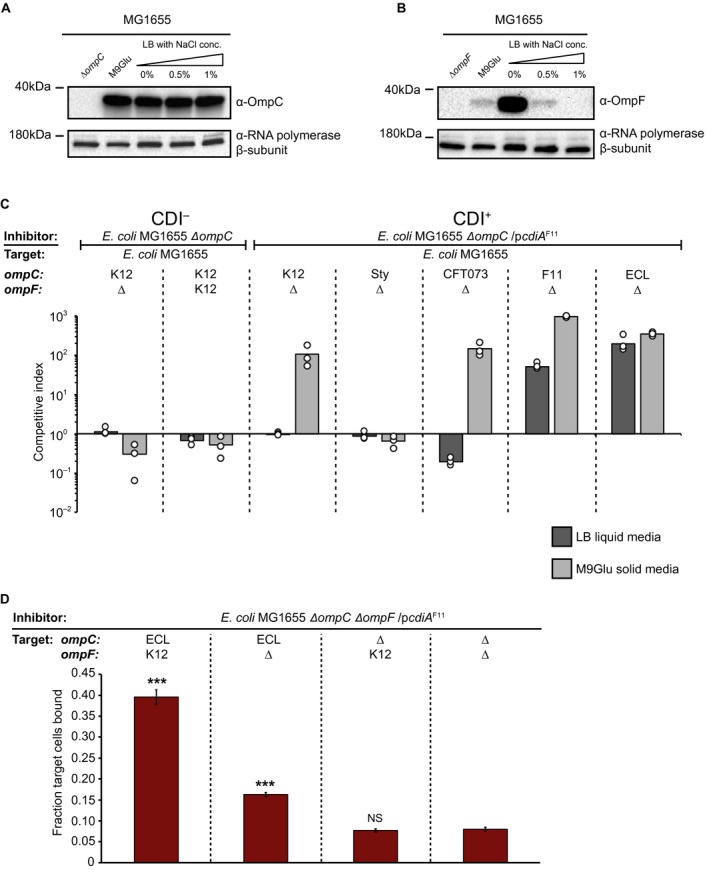
OmpF is not required for class II CdiA mediated growth inhibition. A. OmpC expression from the MG1655 chromosome measured by western blot, probed with an anti‐OmpC antibody. RNA polymerase β‐subunit was used as loading control. B. OmpF expression from the MG1655 chromosome measured by Western blot, probed with an anti‐OmpF antibody. RNA polymerase β‐subunit was used as loading control. C. Average competitive index of cells expressing CdiA^F11^ after co‐culturing with ∆*ompF *MG1655 cells expressing different OmpC’s from the native MG1655 *ompC *locus (*n* = 3 biological replicates). Cells were co‐cultured for 5 h in liquid LB media (dark grey bars) or 24 h on solid M9Glu media (light grey bars). Individual data points of the biological replicates are shown as black and white circles. D. Average fraction of MG1655 target cells with or without *ompC* ^ECL^ and *ompF* ^K12^ bound to CdiA^F11^+ inhibitor cells (*n*=6 biological replicates). Error‐bars are SEM. Statistical significance was determined using a two‐tailed, unpaired student’s *t*‐test where * = *P* < 0.05, ** = *P* < 0.005 and *** = *P* < 0.0005. [Colour figure can be viewed at https://www.wileyonlinelibrary.com]

Another factor that could affect effector delivery, and which could be different on LB than on M9Glu solid media, is the receptor abundance. The expression of the OmpC/OmpF receptor proteins are inversely regulated by osmolarity through a single regulator OmpR, which slightly represses the expression of OmpC and strongly increases the expression of OmpF in response to a decrease in osmolarity (Forst *et al*., 1988). As LB has a higher NaCl concentration (1% NaCl) than M9Glu (0.05% NaCl), less OmpF should be expressed on this media, resulting in less OmpC/OmpF on the cell surface. We therefore investigated if OmpC/OmpF expression differs between LB and M9Glu media by western blot. MG1655 cells expressed very similar levels of OmpC during growth in all tested conditions (Fig. 5A), whereas OmpF expression increased during growth in low salt media (M9Glu or LB with low concentrations of NaCl) (Fig. 5B).

From these results it appeared as if CdiA^F11^ effector delivery could occur even when OmpF levels were very low (undetectable by western blot) (Fig 5B; LB 1% salt is the condition that the competitions in Fig. 1A were performed in), making us question whether OmpF is required for CdiA^F11^ mediated growth inhibition as previously demonstrated (Beck *et al*., 2016). To test if OmpF was required for class II CdiA effector delivery we competed cells expressing CdiA^F11^ with cells expressing K12, *Sty, *CF073*,* F11 or ECL OmpC, in the absence of OmpF. Interestingly, *ΔompF* cells expressing either OmpC^F11^ or OmpC^ECL^ were outcompeted by CdiA^F11^ expressing cells when grown in liquid LB (Fig. 5C), suggesting that OmpF is not required for CdiA^F11^ mediated growth inhibition. In addition, *ΔompF* cells expressing any of the investigated OmpC variants, with the exception of OmpC^Sty^, were inhibited on M9Glu solid media (Fig. 5C), suggesting that OmpF is not essential for class II CdiA effector delivery, but that it is beneficial when the CdiA‐OmpC binding interaction is weak. To investigate if OmpF mediated an increase in relative cell‐cell binding in liquid LB media we measured inhibitor‐target cell interactions between inhibitor cells expressing CdiA^F11 ^and target cells expressing OmpC^ECL^ in the presence or absence of OmpF using a modified cell–cell binding assay. To capture even a weak CdiA‐receptor interaction, inhibitor and target cells were cross‐linked by formaldehyde after 40 min of co‐cultivation. The relative inhibitor‐target cell interactions were measured by flow cytometry as previously described (Fig. 3A and B). A significantly larger fraction (40%) of target cells expressing OmpC^ECL^ and OmpF^K12^ were bound to inhibitor cells expressing CdiA^F11^, as compared to target cells only expressing OmpC^ECL^ (15%) (Fig. 5D). These results further confirm that although OmpF is not essential for cell–cell binding or toxin delivery, it stabilizes the CdiA‐receptor interaction.

### Class II CdiA effector translocation has not evolved to favor intra‐species interactions

**Figure 6:**
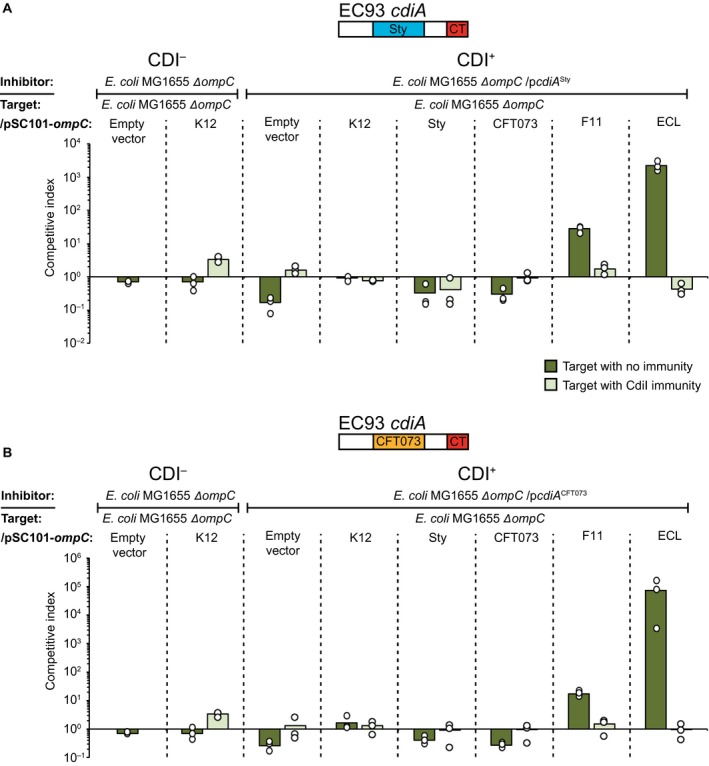
Other Class II CdiA RBD are able to deliver effectors to cells expressing the OmpC receptor from other strains and species. Average competitive index of cells expressing A. CdiA^Sty^ or B. CdiA^CFT073^ after co‐culturing with MG1655 cells expressing different OmpC’s from a constitutive PJ23101 promoter on a low‐copy (pSC101) plasmid with (light green bars) or without (dark green bars) CdiI expressed from a CloDF plasmid (*n* = 3 biological replicates). Cells were co‐cultured for 5 h in liquid LB media. Individual data points of the biological replicates are shown as black and white circles. [Colour figure can be viewed at https://www.wileyonlinelibrary.com]

Our results suggest that CdiA^F11^ has a higher binding affinity and preference for the OmpC^F11^ and OmpC^ECL^ receptors over others. We therefore wanted to investigate if the minor differences observed between the RBD of CdiA^F11^, CdiA^CFT073^ and CdiA^Sty^ (Fig. S1), create an intra‐species preference of receptor binding, i.e. does CdiA^CFT073^ favor a binding to OmpC^CFT073^, and CdiA^Sty^ a binding to OmpC^Sty^? To this end, we created chimeric CdiA proteins where the CdiA receptor‐binding domain of CdiA^F11^ was changed to that of *S. typhi *(CdiA^Sty^) or *E. coli *CFT073 (CdiA^CFT073^ *)*. Cells expressing CdiA^Sty^ did not outcompete cells expressing low levels of OmpC^Sty^ better than cells expressing other OmpC variants in liquid LB media (Fig. 6A). Similarly, cells expressing CdiA^CFT073^ did not outcompete cells expressing low levels of OmpC^CFT073^ better than cells with any other OmpC variant (Fig. 6B), instead both CdiA^Sty^ and CdiA^CFT073^ outcompeted cells expressing OmpC^ECL^ the most (Fig. 6A and B). Furthermore, all target cells expressing any of the different OmpC variants, with the exception of OmpC^Sty^, were inhibited on M9Glu solid media when co‐cultivated with inhibitor cells expressing either CdiA^Sty^ or CdiA^CFT073^ (Fig. S4). Thus, the general trend of growth inhibition for both CdiA^Sty^ and CdiA^CFT073^ is identical to that of CdiA^F11^. Taken together this suggests that the minor differences found in the receptor‐binding domain of different class II CdiA proteins do not result in preferentially targeting of intra‐species receptors.

### Class II CdiA mediated CDI works cross‐species

**Figure 7:**
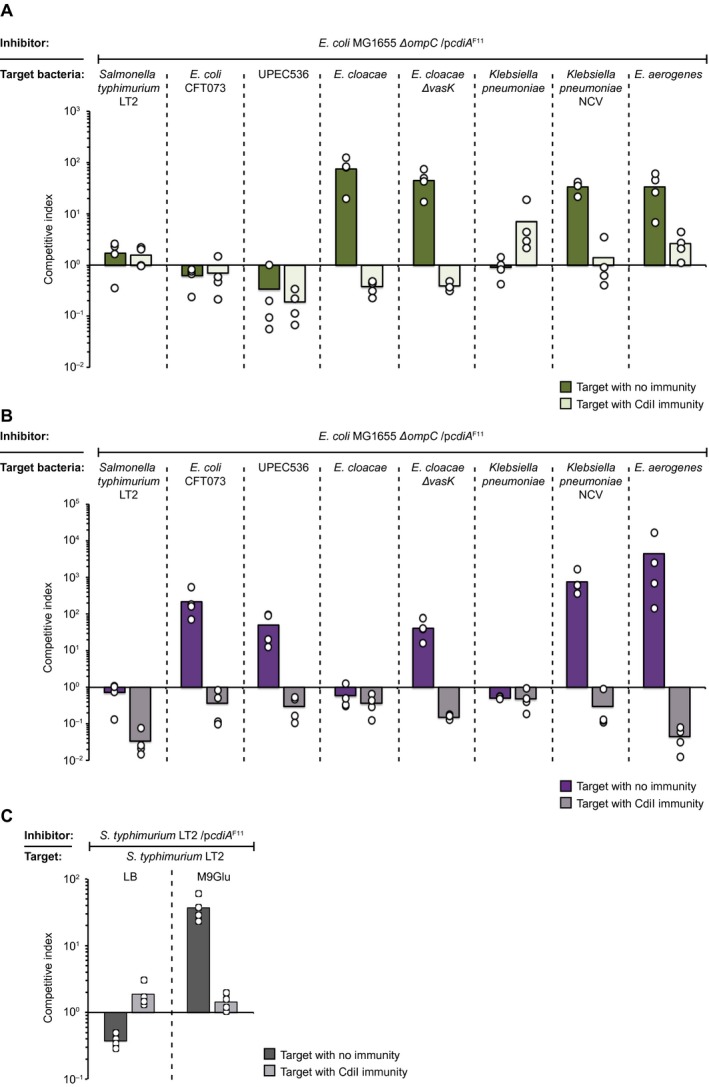
Class II CdiA toxin delivery works cross‐species. A & B. Average competitive index of cells expressing CdiA ^F11^ from a ColE1 plasmid after co‐culturing with *Salmonella typhimurium* *E. coli* CFT073*,* UPEC 536, *Enterobacter* *cloacae*, *Klebsiella pneumoniae *or *Enterobacter aerogenes* with (light green bars) or without (dark green bars) CdiI expressed from a CloDF plasmid*. *Co‐culturing was performed for 5 h in liquid LB media (A) or 24 h on solid M9Glu media (B) (*n* = 4 biological replicates). C. CdiA^F11^ expressing *S. typhimurium* LT2 competed against *S. typhimurium* LT2 with (light grey bars) or without (dark grey bars) CdiI immunity on liquid LB (green) or solid M9Glu (purple) media (*n* = 4 biological replicates). Individual data points of the biological replicates are shown as black and white circles. [Colour figure can be viewed at https://www.wileyonlinelibrary.com]

Our results showed that CdiA mediated growth inhibition of *E. coli* target cells expressing OmpC’s from other species is possible. This does not necessarily infer that *E. coli* cells expressing CdiA^F11^ can actually inhibit the growth of other species. To test if actual strains of *E. cloacae *(ATCC 13047)*,* *S. typhimurium* LT2, UPEC 536 and *E. coli* CFT073 were inhibited by CdiA^F11^ mediated toxin delivery, we competed *E. coli* cells expressing CdiA^F11^ with these bacterial species. In addition, we extended the set of bacteria by including other species where OmpC homologs could be identified bioinformatically (*Enterobacter aerogenes* and *Klebsiella pneumoniae*) (Fig. S5). To control for other factors, e.g. differences in growth or delivery of other toxins that could affect the competition, we provided these bacterial species with CdiI immunity proteins expressed from a medium‐copy plasmid. In liquid LB media, *E. coli* MG1655 cells expressing CdiA^F11^ outcompeted wild‐type *E. cloacae* and *E. aerogenes* cells with 2‐logs, whereas all other tested wild‐type bacterial strains were not outcompeted (Fig. 7A, dark green bars). On M9Glu solid media, *E. coli* MG1655 cells expressing CdiA^F11^ outcompeted *E. coli* CFT073, UPEC 536 and *E. aerogenes* wild‐type cells with 2‐ to 3‐logs (Fig. 7B, dark purple bars), whereas the others were not outcompeted (Fig. 7B). As expected, cells complemented with *cdiI* were not outcompeted on either media (Fig. 7A and B, light colored bars). Interestingly, *E. cloacae* cells were inhibited in liquid LB but not on solid M9Glu media. Previous results suggest that *E. cloacae* harbors a T6SS active against *E. coli* on solid media (Beck *et al*., 2014). Thus, we hypothesized that our CdiA^F11^ expressing *E. coli *could be inhibited back by *E. cloacae* on solid M9Glu media. To test if this was the case we competed *E. coli* MG1655 cells expressing CdiA^F11^ against a ∆*vasK* mutant (unable to form the T6SS) of *E. cloacae. E. coli* MG1655 cells expressing CdiA^F11 ^outcompeted *E. cloacae *cells lacking *vasK* with 2‐logs on both liquid and solid media, suggesting that the ability to compete back could indeed explain the differential inhibition on solid and liquid media. Similarly, *S. typhimurium* cells complemented with *cdiI* outcompeted MG1655 inhibitor cells by 1‐log indicating that *S. typhimurium *cells were indeed inhibited by MG1655 inhibitor cells but that differences in other fitness factors were hiding this fact (Fig. 7B). To normalize against any unknown fitness factors we transformed wild‐type *S. typhimurium* cells with our CdiA^F11^ expressing plasmid and competed these cells against wild‐type *S. typhimurium* (Fig. 7C). *S. typhimurium *cells expressing CdiA^F11^ outcompeted wild‐type *S. typhimurium* by almost 2‐log on M9Glu solid media but not in liquid LB (Fig. 7C). This clearly showed that *S. typhimurium* can both inhibit and be inhibited by a class II CDI system (CdiA^F11^).

Furthermore, no growth inhibition could be observed against *K. pneumoniae* when competed against *E. coli* MG1655 cells expressing CdiA^F11^, whereas *E. aerogenes* was outcompeted on both media (Fig. 7A and B). From the colony morphology it was obvious that the *K. pneumoniae* strain NC105 was forming a lot of capsule (not shown). We therefore examined if *E. coli *cells expressing CdiA^F11 ^were able to outcompete a non‐capsulated mutant of *K. pneumonia.* *E. coli* MG1655 cells expressing CdiA^F11 ^outcompeted capsule deficient *K. pneumoniae* by 2‐log in liquid and on solid media showing that capsule is an obstacle for CdiA^F11^ mediated toxin delivery (Fig. 7A and B).

To verify that effector delivery can occur by OmpC homologs from *K. pneumoniae* and *E. aerogenes*, we cloned the OmpC homologs into the same pSC101 plasmid used to express all other OmpC variants in this study. *E. coli* MG1655 cells expressing CdiA^F11^ outcompeted *E. coli* cells expressing OmpC from *K. pneumoniae* and *E. aerogenes* with 2‐logs (Fig. S6), showing that CdiA^F11^ mediated effector delivery was possible with these OmpC homologs. Taken together, these results suggest that although other factors limit CDI, class II CdiA^F11^ mediated growth inhibition can occur cross‐species.

### CdiA from *Enterobacter cloacae *is an *E. coli* class II CdiA analog

**Figure 8:**
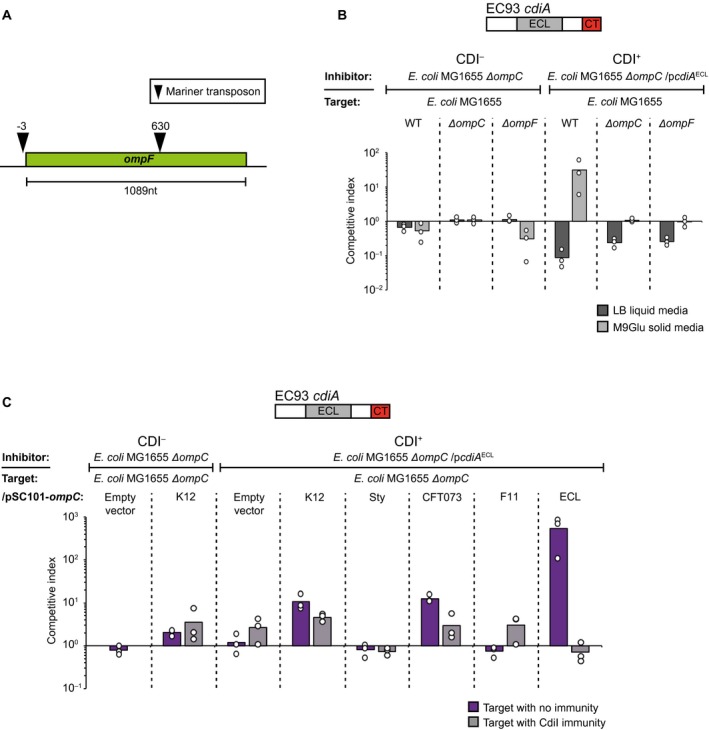
The CdiA protein of *E. cloacae* is a Class II CdiA analog. A. Illustration of the identified mariner transposon insertion sites of CdiA^ECL^ resistant target cells. B & C. Average competitive index of cells expressing CdiA^ECL^ after co‐culturing with MG1655 cells lacking OmpC/F (B) or expressing different OmpC’s from a constitutive PJ23101 promoter on a low‐copy (pSC101) plasmid C. Co‐culturing was for 5 h in liquid LB media (light grey bars) or for 24 h on solid M9Glu (dark grey bars) media (*n* = 3 biological replicates). Individual data points of the biological replicates are shown as black and white circles. [Colour figure can be viewed at https://www.wileyonlinelibrary.com]

This is not the first study showing cross‐species inhibition by CdiA. A previous study looking at CdiA mediated toxin delivery in *E. cloacae* showed that when the CdiA protein of *E. cloacae* (CdiA^ECL^) was artificially over‐expressed from an arabinose inducible promoter, *E. cloacae* strains unable to utilize its T6SS (*∆vasK*) could still inhibit the growth of *E. coli*, suggesting that CDI could work cross‐species (Beck *et al*., 2014)*.* As our results suggest that class II CdiA RBD’s from *E. coli* are promiscuous and capable of delivering CdiA effectors to cells expressing OmpC proteins from other species, we were interested to investigate if the *E. cloacae* CdiA RBD was also promiscuous. The CdiA RBD of CdiA^ECL^ has low sequence homology (56%) to the RBD of CdiA^F11 ^(Fig. S7). We therefore looked for the receptor of the *E. cloacae* CdiA protein by creating a chimeric CdiA protein where the CdiA receptor‐binding domain of CdiA^F11^ was changed to that of *E. cloacae* CdiA (CdiA^ECL^). Next, we created a mariner transposon pool in a MG1655 strain expressing *acrB* (known permissive factor of the EC93 CdiA ionophore toxin used in all our constructs) from a multi‐copy plasmid and enriched for resistant mutants against CdiA^ECL^ by repeatedly competing the transposon pool with MG1655 cells expressing CdiA^ECL^. After three rounds of enrichment, we isolated resistant mutants and used semi‐random arbitrary PCR to identify the insertion sites of the transposons providing resistance toward CdiA^ECL^ toxin delivery. Interestingly, we identified one transposon located 3nt upstream of the *ompF *ORF*,* and one 630 bp in the *ompF* ORF (Fig. 8A), suggesting that OmpF alone or OmpC/OmpF heterotrimers function as the receptor(s) for CdiA^ECL^. To verify that CdiA^ECL ^was also using the OmpC/OmpF heterotrimers as a receptor we competed *∆ompC* or *∆ompF* mutants of *E. coli *MG1655 against inhibitor cells expressing CdiA^ECL^. Cells expressing CdiA^ECL^ outcompeted MG1655 target cells with 1‐log on M9Glu solid media (Fig. 8B), but could not outcompete *E. coli* cells lacking either *ompC *or *ompF *(Fig. 8B), confirming that OmpC/OmpF heterotrimers function as the receptor for CdiA^ECL^.

As CdiA^ECL^ required the same receptor complex as CdiA^F11^, while sharing little sequence homology with CdiA^F11^, we wanted to know if CdiA^ECL^ was an equally promiscuous class II CdiA analog and thus also able to inhibit cells expressing other OmpC receptors*.* To test this we competed cells expressing CdiA^ECL^ against MG1655 cells with *ompC* from *E. cloacae*, K12, Sty, CFT073 or UPEC F11. Inhibitor cells expressing CdiA^ECL^ outcompeted cells expressing low levels of OmpC^ECL ^by >2‐logs, and OmpC^K12 ^or OmpC^CFT073^ by 1‐log on solid M9Glu media (Fig. 8C). Cells expressing low levels of OmpC^F11^ or OmpC*^Sty ^*were not outcompeted by cells expressing CdiA^ECL^ (Fig. 8C) and none of the targets were outcompeted in liquid media (Fig. S8). Taken together, these results show that CdiA^ECL^ is a class II CdiA analog that is also able to deliver effectors cross‐species.

## Discussion

Here, we show that class II CdiA proteins from *E. coli* can allow for efficient effector delivery to a range of *Enterobacteriaceae *spp., including *E. coli*, *S. typhimurium, E. cloacae, E. aerogenes* and *K. pneumoniae*. In contrast to previous findings (Beck *et al*., 2016), our results show that OmpC receptor‐binding by the class II CdiA molecules and their *E. cloacae *CdiA analog is very promiscuous, allowing all four tested CdiA chimeras to deliver effectors cross‐species with little to any intra‐species or strain preference. These findings are supported by previous studies in other species. For example, over‐expression of a naturally occurring CdiBAI system in *E. cloacae* allows inhibition of *E. coli* (Beck *et al*., 2014), which can now be explained by the fact that *E. cloacae* CdiA is a class II analog that uses OmpC as a receptor. Similarly, *Burkholderia pseudomallei* CdiA is able to deliver effectors to the closely related *Burkholdera thailandensis*, probably because the genes involved in synthesizing the CDI receptor (LPS) are similar enough to support cross‐species CDI receptor binding and toxin delivery (Koskiniemi *et al*., 2015).

Broad‐range toxin delivery to other species that share the same growth niche could be beneficial for antagonistic interactions as well as for kin recognition. The ability to recognize siblings from other bacteria is important in any community that exchanges resources. For kin recognition, two types of recognition are important: (i) to identify the own kin from others in order to allow for membrane fusion and exchange of outer membrane components or allow for transfer of proteins only between kin cells, as has been shown to occur by the TraA and IdsD/E systems in *Myxococcus xanthus* and *Proteus mirabilis* respectively (Gibbs *et al*., 2008; Pathak *et al*., 2013). And (ii) inhibition of non‐kin bacteria that would otherwise utilize shared resources, as has been shown to occur by T6SS mediated toxin delivery in *P. mirabilis* or *M. xanthus* (Wenren *et al*., 2013; Vassallo *et al*., 2017). For the latter, very specific protein–protein interactions are favorable and only a few amino acid differences can block binding completely (Cao and wall, 2017). Whereas for the latter, a broader specificity is desirable as it allows inhibition of also more distant unrelated species that could utilize the same resources. CdiA proteins are versatile in that they both allow tight cell–cell binding and toxin delivery (Aoki *et al*., 2005; 2010; Ruhe *et al*., 2015). But how CDI contributes to kin recognition, i.e. if it is by identifying self from others or by inhibiting the growth of other bacteria, is not known. Our results suggest that class II CdiA proteins could function through the latter, where promiscuous effector delivery allows for a broader range of non‐kin recognition and subsequent growth inhibition. However, the impact of such potential broad‐range cross‐species growth inhibition needs to be studied further in natural bacterial communities.

One remaining question is why class II CdiA proteins are different from class I CdiA proteins, where no promiscuous effector delivery can be observed (Ruhe *et al*., 2013b)? Class I and II CdiA molecules bind and recognize different receptor proteins, so a possible explanation could be the abundance of the two receptor‐proteins on the surface of bacterial cells. OmpC is the most abundant protein on the bacterial cell surface, expressed at approximately 100,000 molecules/cell (Schuman, 2006). As a consequence, OmpC proteins are under strong selection to undergo immunogenic variation to avoid targeting by the host immune system (Singh *et al*., 2000; Liu *et al*., 2012; Stenkova *et al*., 2016). In addition, OmpC is the receptor for numerous phages, increasing the selection pressure for constant change to avoid phage infections (Bertozzi Silva *et al*., 2016). This selection for change is easily observable in the extracellular loops of OmpC proteins that vary extensively between different *E. coli* strains (Fig. S2 and S5). Such variations can only be observed between species for BamA, which although also functions as a phage receptor, is expressed more than one order of magnitude less (approximately 4000 molecules/cell (Li *et al*., 2014). Thus, one possibility is that the species‐promiscuity of the class II receptor‐binding domains is a consequence for retaining self‐ or intra‐species delivery, which might be important for the ability to recognize the most important competitors of the own niche, i.e. those very similar but not kin. On the other hand, self‐delivery of CdiA effectors (delivery of toxins to cells with cognate immunity) was recently shown to be important for contact‐dependent signaling and in increasing stress tolerance (Garcia *et al*., 2016; Ghosh *et al*., 2018), suggesting that CDI could be used for both cell–cell communications as well as for antagonistic interactions and kin recognition. Thus, retaining self‐ or intra‐species delivery could be important for functional cell‐cell communication as well as for kin cell recognition.

A final question that arises is if CDI is important in shaping bacterial communities and if so whether it plays a role during initial colonization of a niche and/or in protecting an established micro‐colony. In an aqueous environment with flow (like the host gut), contact‐mediated effector delivery and ability for tight adherence to the targeted cell should be favored over secretion of diffusible toxins to the extracellular milieu, as has been suggested for the glycine zipper like protein toxins from the freshwater bacteria *Caulobacter cresentus* (Garcia‐Bayona *et al*., 2017). Thus, it is possible that a higher binding affinity for certain receptors allows CDI positive cells to adhere and deliver effectors to their main competitors of this niche during initial contact in the host gut and that this is more important for some bacteria than others. For example, we find that CdiA proteins from UPEC 536 and *E. cloacae* have a higher binding affinity to the own receptor over others in our experimental set‐up, but that this is not the case for the CFT073 and *S. typhi* homologs. Once the micro‐colony has been established, however, contacts with new invading species or strains will mainly occur at the edges of the growing colony, a condition that resembles solid media growth. During such growth conditions we observe toxin delivery occurring as efficiently cross‐species as inter‐species. In this context, it is possible that class II CDI functions as a kin recognition system, where antagonistic interactions with a broad range toxin delivery toward other species frequently found in the same niche could potentially allow bacteria with CDI to protect the borders of the growing micro colony against foreign attacks and to restrict the use of shared resources to the own kin. Taken together, our results suggest that class II CdiA proteins are versatile molecular machineries that allow for different behavior depending on the environmental context.

## Experimental procedures

### Strains and growth conditions

The bacterial strains used in this study are listed in Table S1. Strains were grown at 37°C and 200 rpm shaking in Luria‐Bertani broth, LB, (1% Tryptone, 0.5% Yeast extract and 1% NaCl) unless stated otherwise. M9 minimal medium (33.7 mM Na_2_HPO_4_, 22 mM KH_2_PO_4_, 8.55 mM NaCl, 9.35 mM NH_4_Cl, 2 mM MgSO_4_, 0.1 mM CaCl_2_) was supplemented with 0.4% glucose, 0.2% casamino acids and 50 mM FeCl_3_. Media were supplemented with antibiotics when applicable as follows: ampicillin (AMP) 100 µg/ml, chloramphenicol (CAM) 12.5 µg/ml, kanamycin (KAN) 50 µg/ml, streptomycin (STREP) 100 µg/ml and Spectinomycin (SPEC) 50 µg/ml.

### Construction of plasmids and chromosomal constructs

Gene deletions were retrieved from the Keio collection (Baba *et al*., 2006) and moved between strains by P1 transduction. All constructs were verified by PCR and sequencing. For detailed information of the different constructs see the supplementary material.

### Competition assay

The cells were grown overnight in LB. Inhibitor and target cells were mixed at a ratio of 10:1 and either diluted 1:100 in LB for liquid media competitions or 20 µl were spotted on solid M9Glu minimal media. For liquid media competitions, the cells were co‐cultured for 5 h at 37°C with 200 rpm shaking and plated onto LB solid media containing appropriate antibiotics to enumerate inhibitor and target cells as the number of colony‐forming units per milliliter (CFU/ml). For competitions on solid media, the cells were co‐cultured for 24 h at 37°C before suspended in 1×PBS, followed by enumeration of inhibitor and target cells as above. Competitive indexes were calculated as the ratio of inhibitor to target cells at the end of the co‐culture (5 h or 24 h) divided by the ratio at the beginning of the co‐culture. The competitive indexes for three independent experiments are reported ± standard error of the mean.

### Membrane‐protein enrichment and SDS‐PAGE to analyze OmpC

MG1655 cells expressing different variants of OmpC from the chromosome or from a low‐copy plasmid (Table S1) were diluted 1/1000 from an over‐night (ON) culture and grown to stationary phase (OD600 = 2.0). One milliliter of each bacterial culture were pelleted at 21000×*g* for 10 min and re‐suspended in 2 ml of a mild hypotonic lysis buffer (50 mM Tris pH6.8, 1% Triton X‐100, 1 mg/ml lysozyme, 10 mM EDTA, 1 tablet/50 ml SIGMAFAST Protease Inhibitor Cocktail (Sigma‐Aldrich, Germany)) and incubated at room‐temperature for 60 min. Cells were then subjected to six cycles of freeze‐thawing in an ethanol dry‐ice bath before being pelleted at 21000×*g* for 60 min. Supernatants were discarded and pellets was re‐suspended in 2 ml of wash buffer (50 mM Tris pH6.8, 2 mM MgSO_4_, 10 U/ml Benzonase (Sigma‐Aldrich, Germany)) and incubated an additional 20 min on ice to degrade genomic DNA. Cells were then pelleted again at 21000×*g* for 60 min and the pellet was re‐suspended in 100 μl of a 1× membrane‐protein sample buffer (50mM Tris pH6,8, 1% SDS (w/v), 1% Triton X‐100, 10% Glycerol, 0.2% bromophenol blue) and boiled for 5 min at 95°C. 150 mM DTT was then added to each sample followed by the pelleting of membrane debris and undigested genomic DNA at 21000×*g* for 5 min before the supernatant were separated on a Mini‐PROTEAN TGX protein gel (Biorad, USA). Total protein was detected in gel by SYPRO Ruby Protein Gel Stain (Thermo Scientific, USA) and visualized by UV‐table.

### Western blot to analyze OmpC and OmpF

Five hundred microliters of an ON‐culture of MG1655 cells were harvested by centrifugation for 5 min at 21000×*g*. The supernatant was discarded, and cells were re‐suspended in 250 µl of 1× membrane‐protein sample buffer (50mM Tris pH6,8, 1% SDS (w/v), 1% Triton X‐100, 10% Glycerol, 0.2% bromophenol blue) and boiled for 5 min at 95°C. One hundred fifty millimolar DTT was then added to each sample followed by the pelleting of membrane debris and undigested genomic DNA at 21000×*g* for 5 min before the supernatant were separated on a Mini‐PROTEAN TGX gel (Biorad, USA). PageRuler Prestained Protein Ladder (Thermo Scientific, USA) was used as size marker. Proteins were then transferred to a Trans‐Blot Turbo Mini 0.2 µm PVDF membrane (BioRad, USA) using the Trans‐Blot Turbo system (Biorad, USA). OmpC proteins were detected using anti‐OmpC antibody (orb308739, Biorbyt, United Kingdom) and OmpF proteins were detected using anti‐OmpF antibody (orb308741, Biorbyt, United Kingdom). Equal loading was confirmed by probing for RNA polymerase β‐subunit using an anti‐RNAP β‐subunit antibody (ab191598, Abcam, United Kingdom). Secondary antibody toward anti‐OmpC, anti‐OmpF and anti‐RNAP antibodies was anti‐rabbit IgG coupled to HRP (A1949, Sigma‐Aldrich, Germany). Bands were visualized with Clarity Western ECL Substrate (Biorad, USA) and a ChemiDoc MP system (Biorad, USA).

### Flow cytometry cell–cell binding assay without cross‐linking

MG1655 *ΔompC* inhibitor cells with a *galK*::sYFP2‐*cat*R cassette integrated on the chromosome and target cells with a *galK*::dTomato‐*cat*R casett, also integrated on the chromosome (both fluorophores expressed by the synthetic iGEM promoter pJ23101) (Table S1) were diluted 1/100 into LB from an ON‐culture and grown to stationary phase (OD600 = 2.0). Inhibitor and target cells were then mixed at a 5:1 ratio and incubated for 40 min with aeration at 37°C. Samples were then diluted 1/1000 in 1xPBS followed by a gently mixing before samples were analyzed by a MACSQuant VYB flow cytometer using filters B1 (525/50nm) and Y2 (615/20nm) (Miltenyi Biotec). Flow rate was adjusted to allow for 1500 events/sec and at least 100 000 events were collected. Fraction of dTomato‐sYFP2 double events were analyzed in relation to total number of dTomato events (fraction target cells bound to an inhibitor) with FlowJo Software (FlowJo, LLC, USA).

### Flow cytometry cell–cell binding assay with cross‐linking

MG1655 *ΔompC* inhibitor cells with a *galK:*: dTomato‐*cat*R casett integrated on the chromosome and target cells transformed with a plasmid expressing sYFP2 (both fluorophores expressed by the synthetic iGEM promoter pJ23101) (Table S1) were diluted 1/100 into LB from an ON‐culture and grown to stationary phase (OD600 = 2.0). Inhibitor and target cells were then mixed at a 5:1 ratio and incubated for 40 min without agitation at 37°C after which 4% (final conc.) formaldehyde was added and samples were incubate in room‐temp. for an additional 20 min. Samples were then diluted 1/1000 in 1xPBS and vortexed heavily for 10 sec before being analyzed by a MACSQuant VYB flow cytometer, same as above. Flow rate was adjusted to allow for 500 events/sec and at least 25000 events were collected. Fraction of sYFP2‐dTomato double events (fraction target cells bound to an inhibitor) were analyzed in relation to total number of sYFP2 events with FlowJo Software (FlowJo, LLC, USA).

## Conflict of interest

The authors declare no conflict of interest.

## Author contributions

P.V. M.W and S.K conceived the study. P.V. M.W and S.K designed research; P.V. M.W and S.K performed research; P.V. M.W and S.K. analyzed data; P.V. M.W and S.K wrote the paper.
