# Supplementary material for: Class II contact‐dependent growth inhibition (CDI) systems allow for broad‐range cross‐species toxin delivery within the Enterobacteriaceae family
Source: Mol Microbiol. 2019 Mar 18;111(4):1109–25. doi: 10.1111/mmi.14214 (PMC6850196; doi:10.1111/mmi.14214)

Supplementary material for:

Class II Contact-Dependent growth Inhibition (CDI) systems allow for broad-range cross-species toxin delivery within the *Enterobacteriaceae* family

Petra Virtanen[1†], Marcus Wäneskog[1†] and Sanna Koskiniemi[1*]

Affiliations:

[1] Department of Cell and Molecular Biology, Uppsala University, 75124 Sweden.

* Correspondence to: sanna.koskiniemi@icm.uu.se

† These authors contributed equally to this work.

This supplementary file includes:

Supplementary methods

Supplementary data Tables S1-S3

Supplementary references

Supplementary figures S1-S8

Supplementary methods:

Chromosomal and plasmid constructs

Replacement of ompC CDS on the chromosome

A *cat-sacB-amilCP* selection, counter-selection and screening marker, described previously (Nasvall, 2017), was amplified with primers 1190 and 1191 to introduce 40bp overlap at both 5' and 3' ends with homology to the 5' and 3' ends of the *ompC* CDS of MG1655. The *cat-sacB-amilCP* cassette was then integrated onto the genome of MG1655 by lambda red recombination (Datsenko et al., 2000), simultaneously knocking-out *ompC*$^{(MG1655)}$ and generating strain SK2754. *ompC* CDS from *S. typhimurium* LT2, *E. coli* Nissle 1917, *E. coli* F11 and *E. cloacae* ATCC 13047 was amplified with primers 1192 and 1193 and the subsequent PCR products was integrated on the chromosome by lambda red recombination (Datsenko et al., 2000) knocking-out the *cat-sacB-amilCP* cassette and generating seamless *ompC* CDS swaps (SK2777-SK2780). Positive clones were selected for sucrose tolerance and screened for the lack of blue coloring (result of chromo protein AmilCP expression) and chloramphenicol sensitivity. The constructs were verified by PCR using primers 631 and 632, as well as primers 634 and 635, and further verified by sequencing of the PCR products generated from primers 631 and 632.

Construction of pSC101::PJ23101-ompC expressing plasmids

ompC was amplified from *S. typhimurium* LT2, *E. coli* K12 MG1655, *E. coli* Nissle 1917, *E. coli* F11 and *E. cloacae* ATCC 13047 using oligos 606-612 as indicated in Table S3 and inserted into the pSC101 plasmid under the control of the PJ23101 promoter. The resulting plasmids were verified by sequencing using oligos 482 and 483.

Construction of pCloDF1::PJ23101-cdiI^{EC93main} *expression plasmid*

cdiI was amplified from EC93 using oligos 964 and972 as indicated in Table S3 and cloned into the pCloDF1 plasmid under the control of the PJ23101 promoter. The resulting plasmids were verified by sequencing using oligos 986 and 987.

Replacement of the cdiA receptor-binding domains on the medium-copy pcolE1-cdiBAI plasmid

To change the receptor-binding domain of *cdiA*^{EC93}, a *cat-sacB* cassette (Nasvall, 2017) with homology upstream and downstream of the receptor-binding domain of *cdiA*^{EC93} was amplified using oligos 583 and 613. The insert was designed to create SrfI restriction sites upstream and downstream of the receptor-binding domain when inserted. The receptor-binding domains from *E. cloacae* ATCC 13047 and *E. coli* CFT073 *cdiA* was amplified using oligos 794, 795, 584 and 585 and cloned into pDAL660 *cdiA::cat-sacB* using SrfI restriction sites. A potential class II CDI system from *S. typhi* was identified bioinformatically and the receptor-binding domain was synthesized (Gene art, Thermo Scientific, USA) and cloned as described for the other constructs. The resulting plasmids were transformed into *E. coli* K12 MG1655 *ompC::kan* and positive clones were selected for sucrose tolerance and screened for Cam sensitivity, followed by PCR verification and sequencing with primers indicated in table S2. The resulting plasmids carrying the *cdiBAI*^{EC93} system with the receptor binding domain swaps was transformed into *E. coli* K12 MG1655 *ompC::kan*.

sYFP2 reporter plasmid

The *pJ23101-sYFP2* and *osmY-mtagBFP* reporter plasmid (SK2292), previously described in (Ghosh et al., 2018) was modified to remove the ClpXP dependent degradation tags fused to both sYFP2 and mtagBFP. The pSC101 plasmid was amplified with primers 1062 and 1276,

the resulting PCR-products were gel-purified followed by re-circularized with T4 DNA ligase (Thermo Scientific, USA) and transformation into NEB5alfa (New England Biolabs, USA) generating plasmid SK2876. Positive clones were verified by sequencing using the same primers described before (Ghosh et al., 2018). The SK2876 plasmid was amplified with primers 1050 and 1266, the resulting PCR-products were then gel-purified followed by re-circularized with T4 DNA ligase (Thermo Scientific, USA) and transformation into NEB5alfa (New England Biolabs, USA) generating plasmid SK2938. Positive clones were verified same as above.

Table S1. Bacterial strains used in this study

Strain number	Genotype	Origin
SK2	*E. coli* K12 MG1655	(Ghosh et al., 2018)
SK160	*Enterobacter cloacae* ATCC 13047	(Beck et al., 2014)
SK161	*E. coli* UPEC536	Gift from David Low
SK540	*E. coli* CFT073 *cobUS::GFP::cat*	Gift from Agneta Richter
SK620	*E. coli* K12 MG1655 *lacA-cat*	This study
SK1031	*S. typhimurium* LT2 /pDAL7720	This study
SK1323	*E. coli* K12 MG1655 Δ*ompC lacA-cat*	This study
SK1360	*E. coli* K12 MG1655 *ompC::kan* /pDAL7720	This study
SK1435	*E. coli* K12 MG1655 *ompC::kan* /pSK1435	This study
SK1543	*Enterobacter aerogenes* ATCC 13048	This study
SK1735	*K. pneumoniae* subsp. Pneumoniae C105	Gift from Linus Sandegren
SK1736	*K. pneumoniae* subsp. Pneumoniae C105 NCV (non-capsulating mutant)	Gift from Linus Sandegren
SK1998	*E. coli* K12 MG1655 Δ*ompC lacA-cat* /pSC101::*ompC*^{Sty}	This study
SK1999	*E. coli* K12 MG1655 Δ*ompC lacA-cat* /pSC101::*ompC*^{K12}	This study
SK2000	*E. coli* K12 MG1655 Δ*ompC lacA-cat* /pSC101::*ompC*^{CFT073}	This study
SK2001	*E. coli* K12 MG1655 Δ*ompC lacA-cat* /pSC101::*ompC*^{F11}	This study
SK2002	*E. coli* K12 MG1655 Δ*ompC lacA-cat* /pSC101::*ompC*^{ECL}	This study
SK2020	*E. coli* K12 MG1655 Δ*ompC lacA-cat* /pSC101::*ompC*^{Sty} /pSK1752	This study
SK2021	*E. coli* K12 MG1655 Δ*ompC lacA-cat* /pSC101::*ompC*^{K12} /pSK1752	This study
SK2022	*E. coli* K12 MG1655 Δ*ompC lacA-cat* /pSC101::*ompC*^{CFT073} /pSK1752	This study
SK2023	*E. coli* K12 MG1655 Δ*ompC lacA-cat* /pSC101::*ompC*^{F11} /pSK1752	This study
SK2024	*E. coli* K12 MG1655 Δ*ompC lacA-cat* /pSC101::*ompC*^{ECL} /pSK1752	This study
SK2167	*E. coli* K12 MG1655 *ompC::kan* /pSK2167	This study
SK2208	*E. coli* K12 MG1655 *ompC::kan* /pSK2208	This Study
SK2211	*E. coli* K12 MG1655 Δ*ompC lacA-cat* /pSC101::*omp36*$^{E. aerogenes}$	This Study
SK2338	*E. coli* K12 MG1655 Δ*ompC lacA-cat* /pSC101::*ompK36*$^{K. pneuminiae}$	This Study
SK2462	*S. typhimurium* LT2 *STM1553::cat*	Lab collection
SK2468	*E. coli* K12 MG1655 Δ*ompC lacA-cat* /pSC101::*omp36*$^{E. aerogenes}$ /pSK1752	This Study
SK2469	*E. coli* K12 MG1655 Δ*ompC lacA-cat* /pSC101::*ompK36*$^{K. pneuminiae}$ /pSK1752	This Study

SK2565	Eco MG1655 ΔompC ΔompF /pDAL7720	This study
SK2639	*E. coli* K12 MG1655 Δ*ompC lacA-cat* /pSC101 /pSK1752	This study
SK2754	*E. coli* MG1655 *ompC::cat-sacB amilCP* /pSIM5tet	This study
SK2777	*E. coli* MG1655 *ompC* ^{Sty}	This study
SK2778	*E. coli* MG1655 *ompC* ^{CFT073}	This study
SK2779	*E. coli* MG1655 *ompC* ^{F11}	This study
SK2780	*E. coli* MG1655 *ompC* ^{ECL}	This study
SK2817	*E. coli* K12 MG1655 *ompC* ^{Sty} *lacA-cat*	This study
SK2818	*E. coli* K12 MG1655 *ompC* ^{CFT073} *lacA-cat*	This study
SK2819	*E. coli* K12 MG1655 *ompC* ^{F11} *lacA-cat*	This study
SK2820	*E. coli* K12 MG1655 *ompC* ^{ECL} *lacA-cat*	This study
SK3068	*E. coli* K12 MG1655 *lacA-cat* /pCloDF1:: PJ23101-*cdiI*^{EC93}	This study
SK3287	*E. coli* K12 MG1655 *ompF::tet lacA-cat*	This study
SK3288	*E. coli* K12 MG1655 *ompC* ^{Sty} *lacA-cat ompF::tet*	This study
SK3289	*E. coli* K12 MG1655 *ompC* ^{CFT073} *lacA-cat ompF::tet*	This study
SK3362	*E. coli* K12 MG1655 *ompC* ^{F11} *lacA-cat ompF::tet*	This study
SK3363	*E. coli* K12 MG1655 *ompC* ^{ECL} *lacA-cat ompF::tet*	This study
SK3364	*E. coli* K12 MG1655 Δ*ompC* Δ*ompF lacA-cat*	This study
SK3429	*E. coli* K12 MG1655 Δ*ompC* Δ*ompF galK::dTomato-catR* /pDAL7720	This study
SK3430	*E. coli* K12 MG1655 *lacA-cat* /pSK2938	This study
SK3431	*E. coli* K12 MG1655 *lacA-cat ompF::tet* /pSK2938	This study
SK3432	*E. coli* K12 MG1655 *ompC* ^{ECL} *lacA-cat* /pSK2938	This study
SK3433	*E. coli* K12 MG1655 *ompC* ^{ECL} *lacA-cat ompF::tet* /pSK2938	This study
SK3434	*E. coli* K12 MG1655 ΔompC *lacA-cat* /pSK2938	This study
SK3435	*E. coli* K12 MG1655 ΔompC ΔompF *lacA-cat* /pSK2938	This study
SK3792	*E. coli* K12 MG1655 *ompC* ^{Sty} *lacA-cat* /pSK1752	This study
SK3793	*E. coli* K12 MG1655 *ompC* ^{CFT073} *lacA-cat* /pSK1752	This study
SK3794	*E. coli* K12 MG1655 *ompC* ^{F11} *lacA-cat* /pSK1752	This study
SK3795	*E. coli* K12 MG1655 *ompC* ^{ECL} *lacA-cat* /pSK1752	This study
SK3796	*K. pneumoniae* subsp. Pneumoniae C105 /pSK1752	This study
SK3797	*K. pneumoniae* subsp. Pneumoniae C105 NCV /pSK1752	This study
SK3798	*E. coli* UPEC536 /pSK1752	This study
SK3799	*E. coli* K12 MG1655 Δ*ompC lacA-cat* /pSK1752	This study
SK3800	*E. coli* K12 MG1655 Δ*ompC galK::dTomato-catR*	This study
SK3801	*E. coli* K12 MG1655 *galK::dTomato-catR*	This study
SK3802	*E. coli* K12 MG1655 *ompC* ^{Sty} *galK::dTomato-catR*	This study
SK3803	*E. coli* K12 MG1655 *ompC* ^{CFT073} *galK::dTomato-catR*	This study
SK3804	*E. coli* K12 MG1655 *ompC* ^{F11} *galK::dTomato-catR*	This study
SK3805	*E. coli* K12 MG1655 *ompC* ^{ECL} *galK::dTomato-catR*	This study
SK3806	*E. coli* K12 MG1655 Δ*ompC galK::sYFP2-catR* /pDAL7720	This study
SK3807	*E. coli* CFT073 *cobUS::GFP::cat* /pSK1752	This study
SK3808	*S. typhimurium* LT2 *STM1553::cat* /pSK1752	This study
SK3811	*Enterobacter cloacae* ATCC 13047 /pSK1752	This study

SK3812	*Enterobacter cloacae* ATCC 13047 *vasK::kan*	This study
SK3813	*Enterobacter cloacae* ATCC 13047 *vasK::kan* /pSK1752	This study
SK3876	*Enterobacter aerogenes* ATCC 13048 /pSK1752	This study

Table S2. Plasmids used in this study

**Plasmid**	**Genotype**	**Origin**
pDAL660Δ1-39	pWEB::*cdiBAI* ^{EC93}	(Aoki et al., 2005)
pDAL878	pDAL660 derivative, deletion of main CdiA-CT/I	Gift from David Low
pDAL7720	pDAL660Δ1-39 derivative that expresses chimeric CdiA^{EC93} with residues Ser1347 to Gly2205 replaced with residues Ala1345 to Gly2310 from CdiA^{EC536}; Amp^{r}	(Ruhe, 2017)
pCloDF1	Empty vector	
pSC101	Empty vector	
pSK1435	pDAL660Δ1-39 derivative that expresses chimeric CdiA^{EC93} with residues 1208 to 2266 replaced with residues 1225 to 2391 from CdiA$^{\mathit{Eco}\ \mathrm{Nissle}}$; Amp^{r}	This study
pSK1752	pCloDF1:: PJ23101-*cdiI*^{EC93}	This study
SK1978	pMA-T::Salmonella cdiA BD part 1	This study
SK1979	pMK-RQ::Salmonella cdiA BD part 2	This study
pSK1998	pSC101:: PJ23101-*ompC* (*S. typhimurium*)	This study
pSK1999	pSC101:: PJ23101-*ompC* (*E. coli* K12 MG1655)	This study
pSK2000	pSC101:: PJ23101-*ompC* (*E. coli* Nissle 1917)	This study
pSK2001	pSC101:: PJ23101-*ompC* (*E. coli* F11)	This study
pSK2002	pSC101:: PJ23101-*ompC* (*E. cloacae*)	This study
pSK2167	pDAL660Δ1-39 derivative that expresses chimeric CdiA^{EC93} with residues 1208 to 2266 replaced with residues 1224 to 2389 from CdiA$^{\mathit{S.typhi}}$; Amp^{r}	This study
pSK2208	pDAL660Δ1-39 derivative that expresses chimeric CdiA^{EC93} with residues 1208 to 2266 replaced with residues 1326 to 2438 from CdiA$^{\mathit{E.cloacae}}$; Amp^{r}	This study
pSK2292	pSC101(pJ23101:sYFP2(deg-tag) pOsmY:mtagBFP2(deg-tag))	(Ghosh et al., 2018)
pSK2876	pSC101(pJ23101:sYFP2(deg-tag) pOsmY:mtagBFP)	This study
pSK2938	pSC101(pJ23101:sYFP2 pOsmY:mtagBFP)	This study

Table S3. Oligos used in this study

Oligo	Sequence (5' to 3')	Comment
482	GTCTAAGAAACCATTATTAT CATGAC	Forward primer used for sequencing of pSC101 constructs
483	CCAGAACAGCCCGTTTGC	Forward primer used for sequencing of pSC101 constructs
578	CCTGAAGCTGTTTACAGACC GTCTG	Forward primer binding in the bindning domain of *cdiA*(EC93)
579	TCCTTCAGCGTATCCGGCAG	Reverse primer binding in the bindning domain of *cdiA*(EC93)
580	GGTGAATACTGTCAACGGCG GAC	Forward primer binding up-stream of the bindning domain of *cdiA*(EC93)
581	GGCTGTCGCTGGTTTCTTTCG	Reverse primer binding down-stream of the bindning domain of *cdiA*(EC93)
582	GTGGCCACCGGTGATGTCAC ACTGAAACTGATTGCTGCCC gggcatagCAGGAGCTAAGGAA GCTAAAATG	Forward primer to amplify *cat-sacB* cassette with homology upstream and downstream of the *cdiA* receptor binding domain
584	TCACGAATCATGGTACCCTG GC	Forward primer used to amplify the *cdiA* binding domain from *E. coli* CFT073
585	CGGGTCAACCACCACACTGT C	Reverse primer used to amplify the cdiA binding domain from *E. coli* CFT073
606	ATAggatccCAGAGGGTTAATA ACATGAA	Forward primer used to amplify *ompC* from *E. coli* MG1655, *S. Typhimurium* LT2, *E. coli* F11 or *E. coli* CFT073 and *E. cloacae* ATCC 13047 introducing restriction site for BamHI at the 5' end
608	GCGgtcgacCAATCGAGATTAG AACTGG	Reverse primer to amplify *ompC* from *E. coli MG1655*, introducing restriction site for SalI at the 3' end
609	GCGgtcgacCTGATTAGAACTG GTAAACC	Reverse primer to amplify *ompC* from *S. Typhimurium* LT2, introducing restriction site for SalI at the 3' end
610	GCGgtcgacAAAGCGATTAGAA CTGGT	Reverse primer to amplify *ompC* from *E. cloacae* ATCC 13047, introducing restriction site for SalI at the 3' end
611	GCGgtcgacCGAGATTAGAACT GGTAAAC	Reverse primer to amplify *ompC* from *E. coli* F11, introducing restriction site for
612	GCGgtcgacGAGATTAGAACTG GTAAACC	Reverse primer to amplify *ompC* from *E. coli* CFT073, introducing restriction site for SalI at the 3' end
613	GTGGCCACCGGTGATGTCAC ACTGAAACTGATTGCTGCCC gggcatagTGTAGGCTGGAGCTG CTTCGA	Forward primer to amplify *cat-sacB* cassette with homology upstream and downstream of the *cdiA* receptor binding domain
631	CGTGATTATCCTCATGC	Forward primer binding up-stream of *ompC* of MG1655

632	TTAGTATCATATTCGTGTTGG	Reverse primer binding down-stream of *ompC* of MG1655
634	GAAACTCAGGTTACTGACCAGC	Forward primer binding in the *ompC* of MG1655
635	GAACTGGTACTGAGCAACAGC	Reverse primer binding in the *ompC* of MG1655
665	CGGAACAACCACTGCACA	Primer to be used for sequencing of *S. Typhi*, *E. coli* CFT073 and F11 *cdiA* binding domain swaps
666	CTGAATGCGGGGCTGATAAG	Primer to be used for sequencing of *S. Typhi*, *E. coli* CFT073 and F11 *cdiA* binding domain swaps
667	GAGACAGTCCGTCAGCAG	Primer to be used for sequencing of *S. Typhi, E. coli* CFT073 and F11 *cdiA* binding domain swaps
679	CAACGGACTGGGTTCAGACAC	Primer to be used for sequencing of *S. Typhi, E. coli* CFT073 and F11 *cdiA* binding domain swaps
779	GACCTGCTGGTGAATTAC	Forward primer that binds upstream of the binding domain in *cdiA* EC93 (pDAL660)
780	GTATCTGATTACGGTGAACC	Forward primer that binds in conserved region in *cdiA* binding domain from *E. coli* CFT073, F11 and *E. cloacae* ATCC 13047.
781	GGTTCACCGTAATCAGATAC	Reverse primer that binds in conserved region in *cdiA* binding domain from *E. coli* CFT073, F11 and *E. cloacae* ATCC 13047.
786	CATCTGTTCCGTGTCTGAACCCA	Reverse primer used together with 584 to amplify first part of synthesized *S. Typhi cdiA* binding-domain
787	TGGGTTCAGACACGGAACAGATG	Forward primer used together with 585 to amplify second part of synthesized *S. Typhi cdiA* binding-domain
794	TCGTTAACACCGGCACCCT	Forward primer to amplify *cdiA* binding domain from *E. cloacae* ATCC 13047
795	TGGCTCAATCACCACGCTG	Reverse primer to amplify *cdiA* binding domain from *E. cloacae* ATCC 13047
796	AGCAAATGGGAGCCTCTGAC	Forward primer for sequencing of *E. cloacae cdiA* binding domain
835	GCATTCAGACTCAGGTCGTT	Reverse primer for sequencing of *S. Typhi cdiA* binding domain
838	GTCAGAGGCTCCCATTTGCT	Reverse primer primer for sequencing of *E. cloacae cdiA* binding domain
839	AACGAGATTGTGACCAGCGA	Forward primer for sequencing of *E. cloacae cdiA* binding domain
964	ATATGTCGACGGTAATAAGGAAGGGGC	Forward primer to amplify cdiI^{EC93main} with SalI restriction-site
972	TTAAGTCGACCTATTTCTGTCTAAGATACTAAGGC	Reverse primer to amplify *cdiI*^{EC93main} with SalI restriction-site

986	ATCACCACCACCATCACGTG G	Primer to be used for sequencing of pCloDF::*cdiI*^{EC93main} plasmid
987	GTGACCGTGTGCTTCTCAAA TGC	Primer to be used for sequencing of pCloDF::*cdiI*^{EC93main} plasmid
1050	CCCTAGACCTAGGGTACGG	Forward primer for amplifying pSC101 plasmid backbone
1062	CTGCTCGAGGTGAAGACGAA AGGG	Reverse primer for amplifying pSC101 plasmid backbone
1190	GCAAATAAAGGCATATAACA GAGGGTTAATAACATGAAAG GATCTATCAACAGGAGTCCA AGC	Forward primer used to amplify *cat-sacB-amilCP* casette with homology to up-stream sequence of *ompC* CDS of MG1655
1191	TGTTCGATATCAATCGAGAT TAGAACTGGTAAACCAGACC GTGTAGGCTGGAGCTGCTTC	Reverse primer used to amplify *cat-sacB-amilCP* casette with homology to down-stream sequence of *ompC* CDS of MG1655
1192	GCAAATAAAGGCATATAACA GAGGGTTAATAACATGAAAG	Forward primer used to amplify *ompC* CDS from *S. Typhimurium* LT2, *E. coli* F11, *E. coli* CFT073 and *E. cloacae* ATCC 13047
1193	TGTTCGATATCAATCGAGAT TAGAACTGGTAAACCAGACC	Reverse primer used to amplify *ompC* CDS from *S. Typhimurium* LT2, *E. coli* F11, *E. coli* CFT073 and *E. cloacae* ATCC 13047
1262	GGTGAATTACCACACATTCA GC	Forward primer used to amplify *cdiA* BD^{UPEC536}
1263	TCGTCTCAGACTTCGACTGC	Forward primer used to amplify *cdiA* BD^{UPEC536}
1264	GCAGGCAACCTGCTGCTTGA CGCTCAGGACTTCAGTGGTC CATATGAATATCCTCCTTAG TTCC	Forward primer used to amplify *cat-sacB-amilCP* casette with homology to up-stream sequence of *cdiA* BD^{EC93}
1265	GGTGACCGCATTATTGATGG CACTACCCACCGCACCGGAC GTAGGCTGGAGCTGCTTC	Reverse primer used to amplify *cat-sacB-amilCP* casette with homology to up-stream sequence of *cdiA* BD^{EC93}
1266	TTATTTATACAGCTCATCCAT ACCC	Reverse primer to amplify sYFP2 without degradation tag
1276	TTAATTCAGTTTATGACCCA GCTTGCTAG	Forward primer to amplify mtagBFP without degradation tag

References:

Aoki, S. K., Pamma, R., Hernday, A. D., Bickham, J. E., Braaten, B. A., & Low, D. A. (2005). Contact-dependent inhibition of growth in Escherichia coli. *Science, 309*(5738), 1245-1248. doi:10.1126/science.1115109

Beck, C. M., Morse, R. P., Cunningham, D. A., Iniguez, A., Low, D. A., Goulding, C. W., & Hayes, C. S. (2014). CdiA from Enterobacter cloacae delivers a toxic ribosomal RNase into target bacteria. *Structure, 22*(5), 707-718. doi:10.1016/j.str.2014.02.012

Datsenko, K. A., & Wanner, B. L. (2000). One-step inactivation of chromosomal genes in Escherichia coli K-12 using PCR products. *Proceedings of the National Academy of Sciences of the United States of America, 97*(12), 6640-6645. doi:10.1073/pnas.120163297

Ghosh, A., Baltekin, O., Waneskog, M., Elkhalifa, D., Hammarlof, D. L., Elf, J., & Koskiniemi, S. (2018). Contact-dependent growth inhibition induces high levels of antibiotic-tolerant persister cells in clonal bacterial populations. *EMBO J.* doi:10.15252/embj.201798026

Nasvall, J. (2017). Direct and Inverted Repeat stimulated excision (DIRex): Simple, single-step, and scar-free mutagenesis of bacterial genes. *PLoS One, 12*(8), e0184126. doi:10.1371/journal.pone.0184126

Ruhe, Z. C., Nguyen, J. Y., Xiao, J., Koskiniemi, S., Beck, C. M., Perkins, B., Low, D. A., Hayes, C. S. (2017). CdiA effectors use modular receptor-binding domains to recognize target bacteria. *Submitted manuscript.*

Supplementary figures:

Figure S1. Alignment of CdiA receptor binding domains from *Escherichia coli* strains UPEC 536, UPEC F11, CFT073 and *Salmonella typhi*. The receptor binding domains located between amino acids 1300-2390 in CdiA are shown. Homologous residues are shown in grey and non-homologous residues are white.

Figure S1

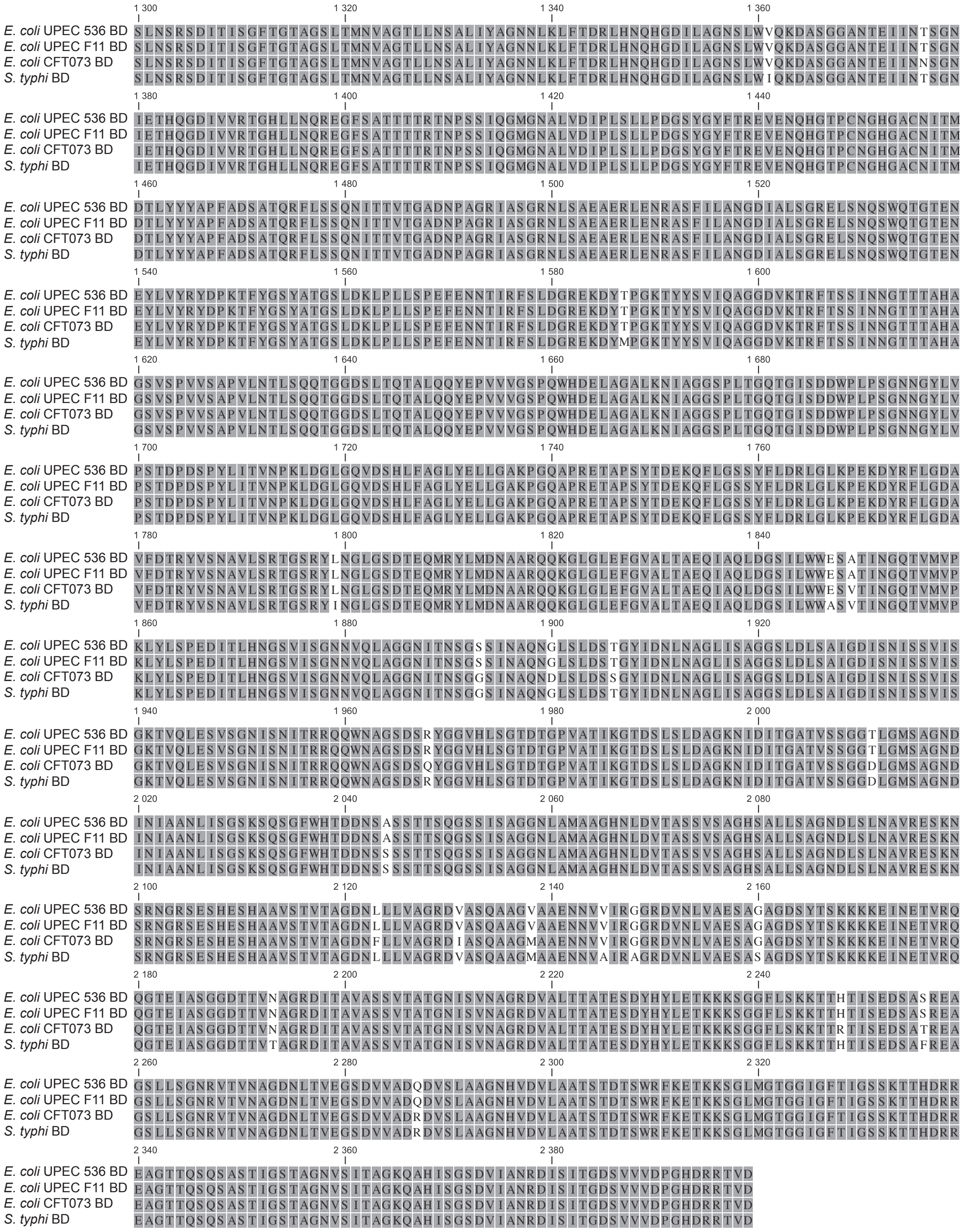

Figure S2. Alignment of OmpC proteins from *Escherichia coli* strains K12 MG1655, UPEC 536, UPEC F11, CFT073 and *Enterobacter cloacae and Salmonella typhi.* Homologous residues are shown in grey and non-homologous residues are white. The location of the 8 extracellular loops in OmpC are marked with a black arrow above the sequence.

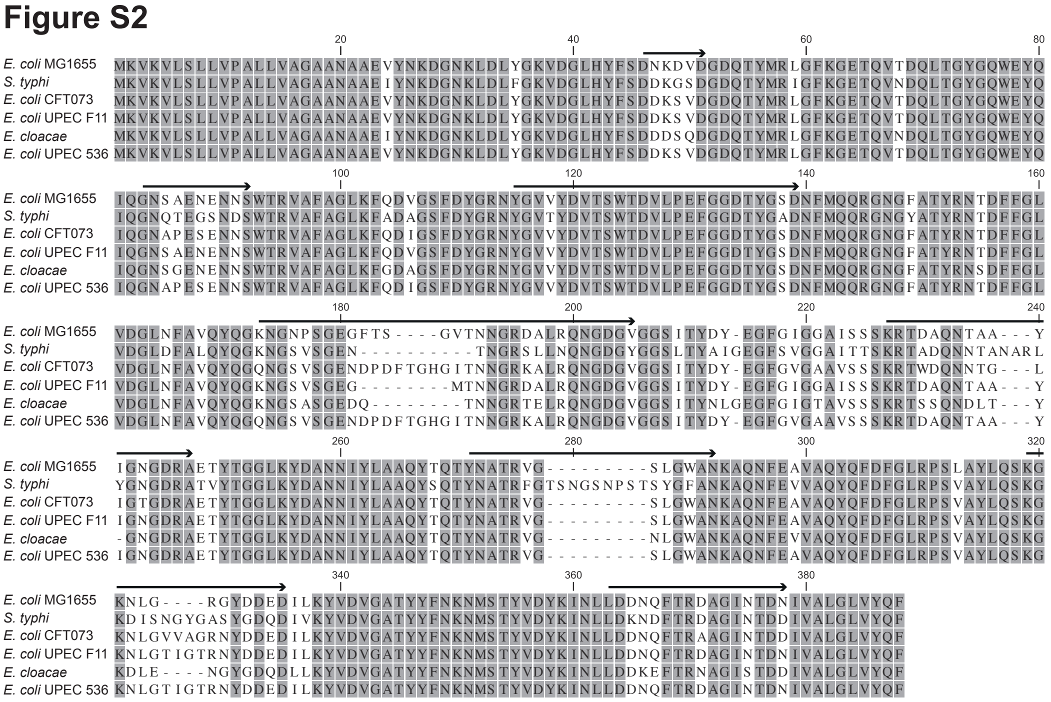

Figure S3. Correlation of cell-cell binding with growth inhibition. Fraction of target cells, expressing different OmpC's, bound to inhibitor cells expressing $CdiA^{F11}$ was correlated against the measured competitive index of the same strains when co-cultured in LB (n=3 biological replicates). Statistical significance was determined using Pearson correlation tables for R^2 values.

Figure S3

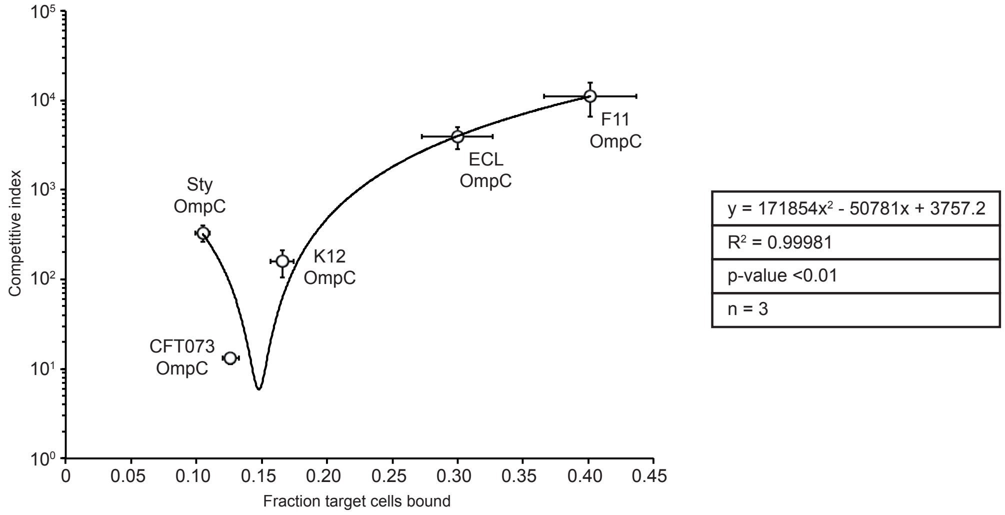

Figure S4. Other Class II CdiA RBD is able to deliver effectors to cells expressing the OmpC receptor from other strains and species on solid media. A & B) Average competitive index of cells expressing CdiA^{Sty} (**A**) or CdiA^{CFT073} (**B**) after co-culturing with MG1655 cells expressing different OmpC's from a low-copy (pSC101) plasmid with (light grey bars) or without (dark purple bars) CdiI expressed from plasmid (n=3 biological replicates). Cells were co-cultured for 24h on solid M9Glu media. Individual data points of the biological replicates are shown as black and white circles.

Figure S4

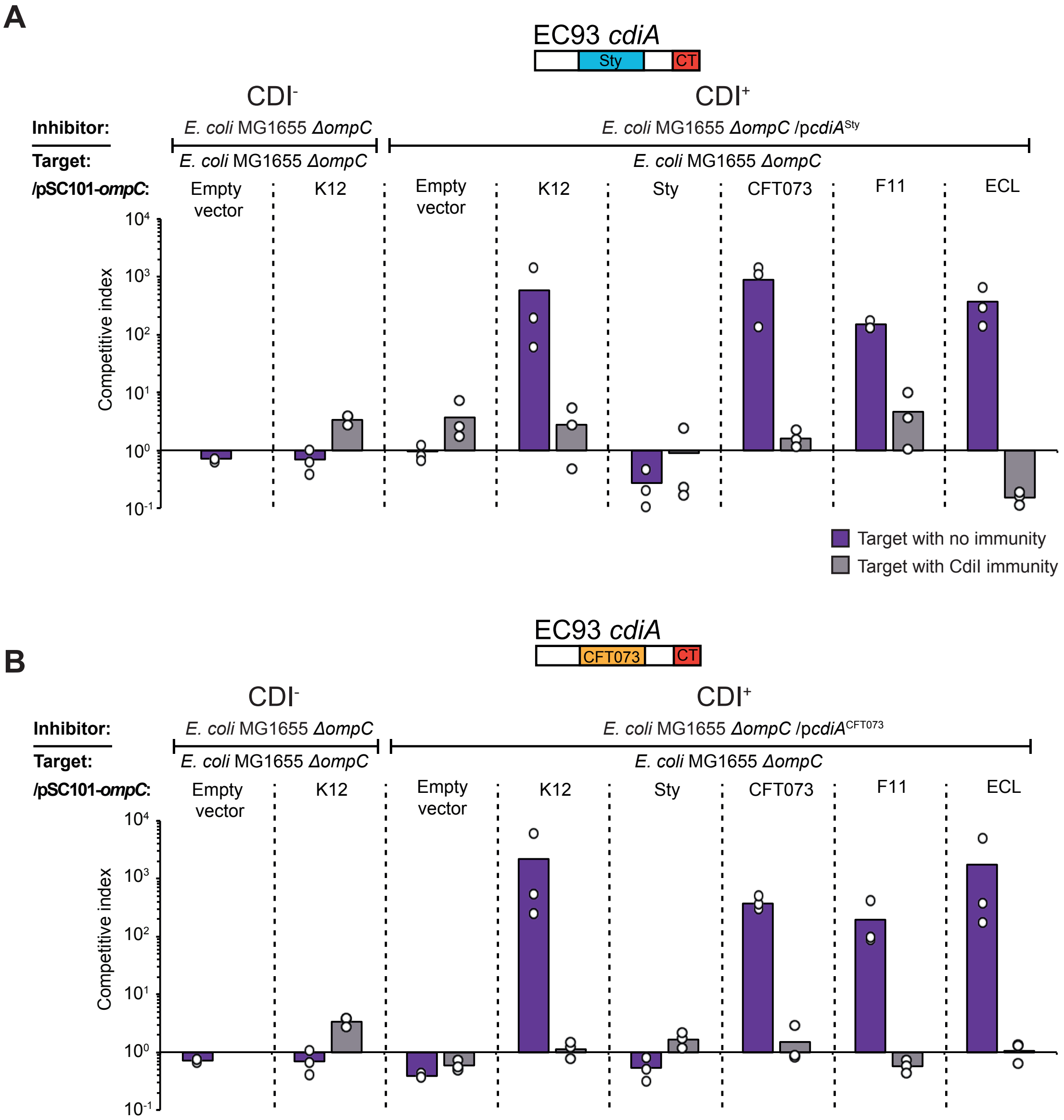

Figure S5. Alignment of OmpC homologs from *Klebsiella pneumoniae* and *Enterobacter aerogenes*. Homologous residues are shown in grey and non-homologous residues are white.

Figure S5

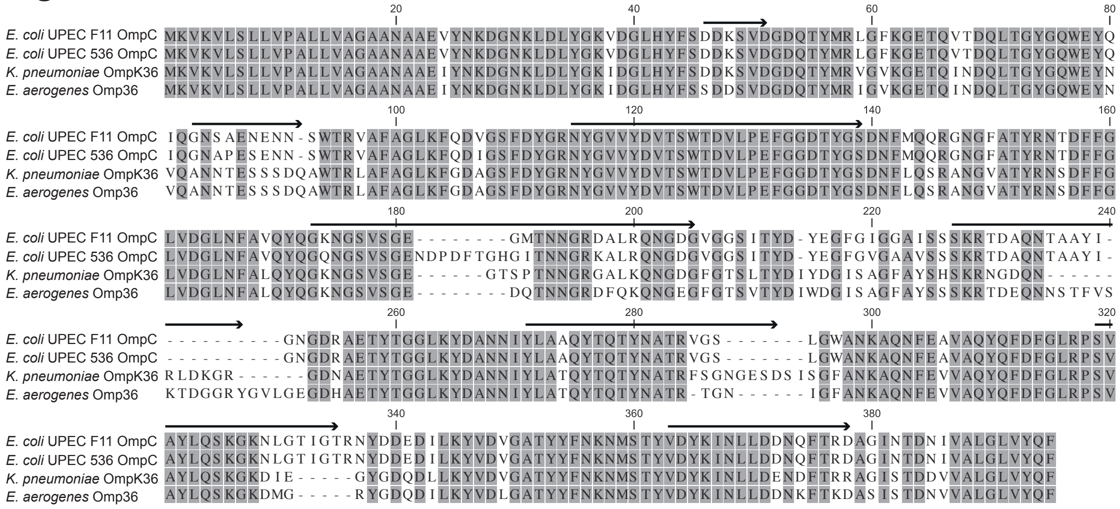

Figure S6. Expression of OmpC homologs from *Klebsiella pneumoniae* and *Enterobacter aerogenes* allow effector delivery into MG1655 cells. Average competitive index of cells expressing CdiA^{F11} (after co-culturing with MG1655 cells expressing different OmpC's from a low-copy (pSC101) plasmid with (light grey bars) or without (dark purple bars) CdiI expressed from plasmid (n=3 biological replicates). Cells were co-cultured for 24h on solid M9Glu media. Individual data points of the biological replicates are shown as black and white circles.

Figure S6
A

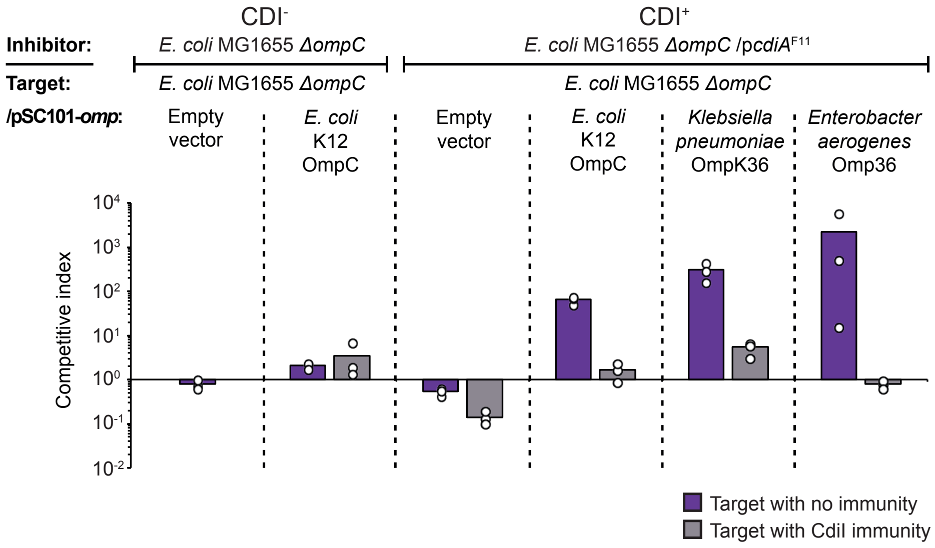

Figure S7. Alignment of CdiA receptor-binding domains from *Escherichia coli* UPEC F11 and *Enterobacter cloacae*. Homologous residues are shown in grey and non-homologous residues are white.

Figure S7

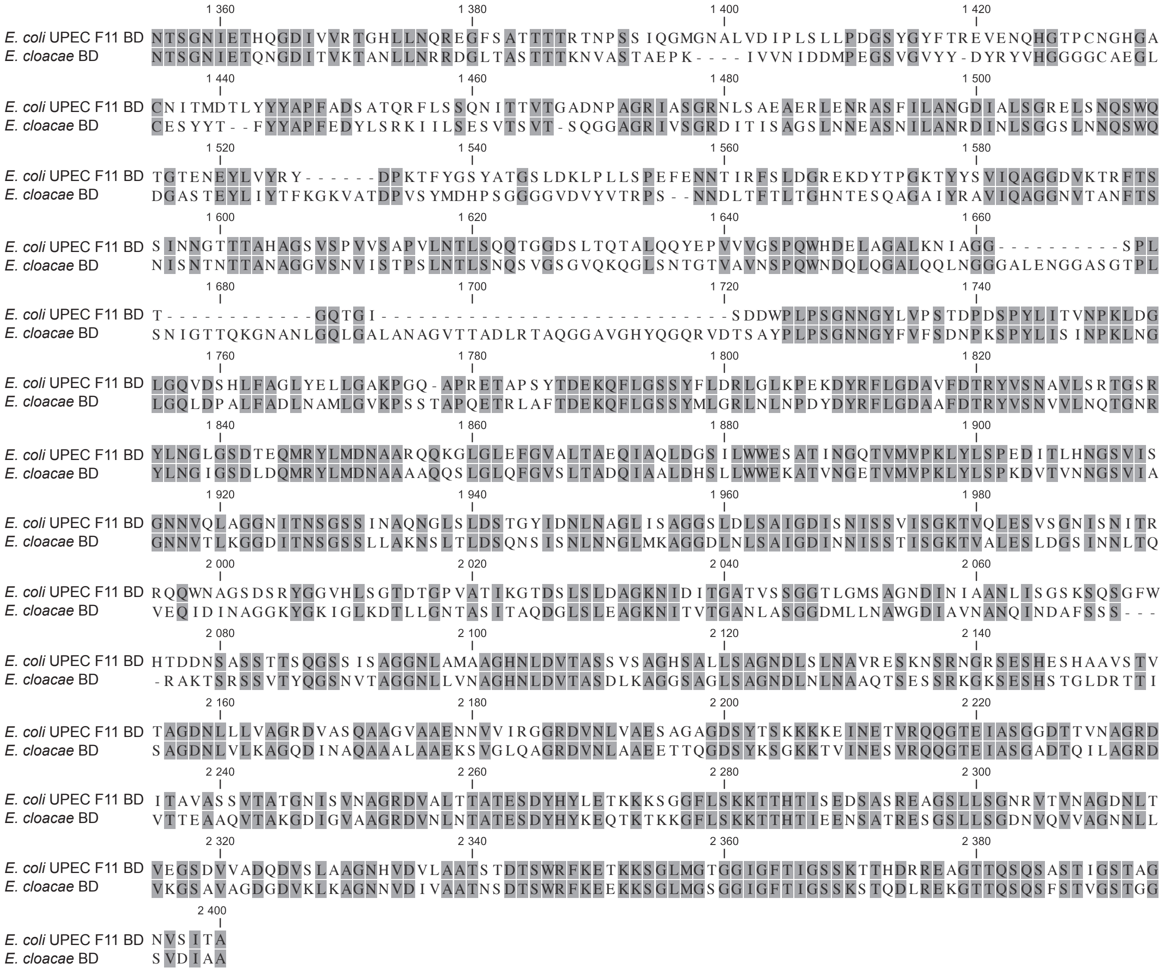

Figure S8. The CdiA protein of *E. cloacae* does not inhibit *E. coli* MG1655 expressing different OmpC's in liquid media.

Average competitive index of cells expressing CdiA^{ECL} after co-culturing with MG1655 cells expressing different OmpC's from a constitutive PJ23101 promoter on a low-copy (pSC101) plasmid. Co-culturing was for 5h in liquid LB media with (light green bars) or without (dark green bars) CdiI expressed from plasmid (n=3 biological replicates). Individual data points of the biological replicates are shown as black and white circles.

Figure S8

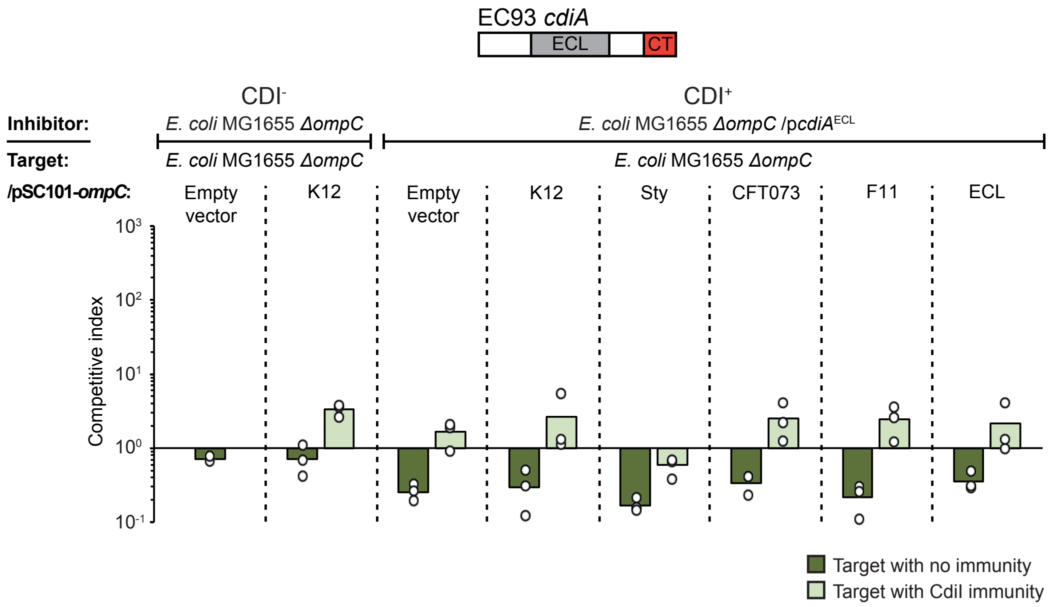

Supplement: Supplementary file 1 [file MMI-111-1109-s001.pdf]
